# Non-destructive orientation tracking of individual β-Sn grains in die-attach solder joints

**DOI:** 10.1107/S1600577526001475

**Published:** 2026-03-20

**Authors:** Jaemyung Kim, Yujiro Hayashi, Hiroaki Tatsumi, Hiroshi Nishikawa, Makina Yabashi

**Affiliations:** a RIKEN SPring-8 Center, 1-1-1 Kouto, Sayo-cho, Sayo-gun, Hyogo679-5148, Japan; b Joining and Welding Research Institute Osaka University 11-1 Mihogaoka Ibaraki Osaka567-0047 Japan; Brazilian Synchrotron Light Laboratory, Brazil

**Keywords:** *i*-S3DXRD, orientation mapping, thermal cycling, X-ray orientation lamino­graphy

## Abstract

Understanding the failure mechanisms of Pb-free solder joints during thermal cycling is a critical issue in electronic packaging, where solder joints strongly influence device reliability and lifetime. While electron backscatter diffraction (EBSD) has been widely used for this purpose, its destructive nature prevents tracking the microstructural evolution of solder joints from their pristine state. Non-destructive X-ray-based orientation microscopy techniques can address this limitation but typically require cylindrical, pillar-shaped specimens, which are incompatible with the planar geometry of practical solder joints. To overcome this constraint, we apply inclined scanning three-dimensional X-ray diffraction microscopy (*i*-S3DXRD), which is specifically designed for plate-like specimens. Using *i*-S3DXRD, we found that thermal cycling induces preferential reorientation of β-Sn grains toward specific crystallographic directions relative to the Cu substrate. This reorientation is associated with recrystallization-assisted grain boundary migration and grain coalescence, and is closely related to the thermal fatigue behavior of Pb-free solder joints.

## Abstract 2

This study introduces inclined scanning three-dimensional X-ray diffraction microscopy (*i*-S3DXRD) that enables three-dimensional orientation mapping of planar polycrystalline materials, revealing the thermal fatigue mechanism of Pb-free solder joints.

## 1. Introduction

Polycrystalline alloys and their joints are ubiquitous in industries such as infrastructure, transportation, aerospace, and security. Failures in these materials, often caused by the accumulation of fatigue and damage (Pineau *et al.*, 2016 ▸), can have catastrophic consequences. Not only for controlling the lifetime of products but also for ensuring safety, understanding the underlying failure mechanism of the material is a key issue in the industry. However, the failure mechanisms are complex and not fully understood, as they are influenced by various factors including grain size (Lasalmonie & Strudel, 1986 ▸), morphology (Boyce *et al.*, 2013 ▸), and crystallographic orientation (Lu *et al.*, 2008 ▸).

Traditionally, the failure mechanisms of polycrystalline materials have been investigated through microstructural observation using electron backscatter diffraction (EBSD) (Adams, 1997 ▸; Humphreys, 2001 ▸; Wright *et al.*, 2011 ▸). In EBSD, grain orientation maps are reconstructed by analyzing Kikuchi patterns in a pixel-by-pixel manner, and the method has become a standard tool in microstructural analysis. EBSD is typically combined with scanning electron microscopy (SEM), which enables the simultaneous acquisition of both morphological information and orientation maps. More recently, the development of tri-beam microscopy—combining a femtosecond (fs) laser, a focused ion beam (FIB) and an electron beam (e-beam)—has made three-dimensional microstructural analysis possible (Gholinia *et al.*, 2024 ▸). In this approach, rapid surface ablation by the fs-laser and precise milling by the FIB expose fresh cross-sections, while SEM imaging and EBSD measurements are performed simultaneously. However, studying the evolution, changes, and degradation of microstructures within the bulk and the buried interfaces remains challenging due to the destructive nature of the technique (MacSleyne *et al.*, 2009 ▸; Rowenhorst *et al.*, 2010 ▸). Therefore, there is a strong demand for non-destructive orientation microscopy techniques capable of probing deep inside materials.

The failure mechanism can also be understood in a non-destructive way by tracking the microstructural changes at the same positions of the same specimen. Because destructive methods alter the original conditions of the specimen, the behavior of the artificially manipulated surface does not represent its bulk behavior. For example, the grains in the mechanically polished free surface can shear freely in the out-of-plane direction under tensile strain, known as a ‘floating grain’ effect, which does not represent the bulk property (Mayo & Nix, 1989 ▸). These surface grains may not deform, retain their initial shapes, and drift under tensile strain (Alabort *et al.*, 2016 ▸). Therefore, the microstructure of the material should be investigated in non-destructive ways to avoid such ambiguity.

For the non-destructive failure analysis of devices, scanning acoustic microscopy (SAM), which employs ultrasound, has been widely used (Yazdan Mehr *et al.*, 2015 ▸). However, this technique offers limited spatial resolution and does not provide crystallographic information. Similarly, while X-ray microtomography enables the visualization of fatigue-induced crack evolution (Regalado *et al.*, 2019 ▸), it also lacks crystallographic insight. Computed tomography (CT), which combines multiple X-ray images from different angles to create a 3D reconstruction of the internal structure, is widely used for the non-destructive observation of the material (Kalender, 2006 ▸; Cnudde & Boone, 2013 ▸). However, when the sample has a plate-like geometry, CT often produces noisy or severely degraded reconstructions. If the long side of the specimen is parallel to the X-ray beam at certain angles, it causes excessive X-ray absorption. As a result, the intensity of the transmitted X-rays goes down to the detection limit, which hampers the CT image reconstruction.

One solution to avoid excess X-ray absorption is to incline the rotation axis relative to the X-ray beam direction. In this rotation geometry, excessive X-ray absorption is automatically avoided, making it easier to detect transmitted X-rays at any rotation angle with a constant X-ray attenuation, which is known as computerized lamino­graphy (CL) (Gondrom *et al.*, 1999 ▸; Hoshino *et al.*, 2011 ▸). Due to its unique rotation geometry, CL is widely used in the inspection of plate-like products such as ball grid array (BGA) on printed circuit boards (PCBs) (Moore *et al.*, 2002 ▸).

Although CT and CL can observe the inside of the material in non-destructive ways, they are not sensitive to the crystallographic orientation of grains in polycrystals (Ludwig *et al.*, 2009 ▸). Therefore, non-destructive orientation microscopy techniques sensitive to grain orientation, such as X-ray-based methods, are required. Several approaches have been developed for this purpose, including differential aperture X-ray microscopy (DAXM) (Larson *et al.*, 2002 ▸; Levine *et al.*, 2006 ▸; Barabash *et al.*, 2009 ▸; Ice *et al.*, 2011 ▸; Larson & Levine, 2013 ▸), diffraction contrast tomography (DCT) (Johnson *et al.*, 2008 ▸; Ludwig *et al.*, 2008 ▸; Reischig *et al.*, 2013 ▸; Yi *et al.*, 2015 ▸; Oddershede *et al.*, 2019 ▸; Sun *et al.*, 2024 ▸), high-energy diffraction microscopy (HEDM) (Bernier *et al.*, 2011 ▸; Lienert *et al.*, 2011 ▸; Li *et al.*, 2012 ▸; Pokharel *et al.*, 2014 ▸), 3D X-ray diffraction microscopy (3DXRD) (Margulies *et al.*, 2001 ▸; Poulsen *et al.*, 2001 ▸; Poulsen *et al.*, 2003 ▸; Poulsen & Fu, 2003 ▸; Poulsen, 2012 ▸; Sharma *et al.*, 2012 ▸; Schmidt, 2014 ▸; Winther *et al.*, 2017 ▸; Juul *et al.*, 2017 ▸; Juul *et al.*, 2020 ▸), and scanning 3D X-ray diffraction microscopy (S3DXRD) (Hayashi *et al.*, 2015 ▸; Hayashi *et al.*, 2019 ▸; Hektor *et al.*, 2019 ▸; Henningsson *et al.*, 2020 ▸; Kim *et al.*, 2023*a* ▸; Henningsson *et al.*, 2024 ▸). Recent studies have demonstrated complementary approaches for extracting local texture information in cases where severe diffraction-peak overlap precludes grain-resolved indexing, which require sample rotation under an inclined geometry (Carlsen *et al.*, 2024 ▸; Frewein *et al.*, 2024 ▸).

Except for DAXM, dedicated to the grain-resolved orientation map of the flat surface under polychromatic X-ray beam illumination, most of the aforementioned X-ray-based methods require sample rotation around the rotation axis perpendicular to the incident X-ray beam. In that sample rotation geometry, specimens are limited to small cylindrical pillars to avoid excessive X-ray absorption. However, many practical products, such as PCBs, steel sheets, solder joints with BGA, and die-attach solder joints for power modules as electronic packaging applications, have the form of plate-like shapes with high aspect ratios.

Unlike other techniques that rely on monochromatic X-rays, the recently developed inclined scanning three-dimensional X-ray diffraction microscopy (*i*-S3DXRD) (Kim *et al.*, 2023*b* ▸) employs an inclined rotation geometry similar to that used in lamino­graphy. That study demonstrated the method using an α-Fe wire specimen and further showed the feasibility of sub-voxel analysis in 3D. However, its applicability to plate-like samples has not yet been confirmed.

Die-attach Pb-free solder joints are a representative example of such planar components for high-temperature applications (Manikam & Cheong, 2011 ▸). These joints play a critical role in electronic packaging by providing both electrical interconnection and mechanical support. They are primarily composed of tin (Sn) as the base material, with various additives such as bis­muth (Bi), copper (Cu), indium (In), zinc (Zn), antimony (Sb), silver (Ag), nickel (Ni), and chromium (Cr), among others (Cheng *et al.*, 2017 ▸). These additive elements influence key properties—including melting temperature, wettability, and thermal fatigue resistance—making each composition suitable for specific applications.

Among lead-free solder alloys, SAC (Sn–Ag–Cu), Sn–Ag, and Sn–Cu are particularly popular due to their well balanced combination of mechanical strength, thermal reliability, and soldering performance. However, due to the high cost of Ag, manufacturers are increasingly shifting toward Sn–Cu solders as an alternative (Cheng *et al.*, 2017 ▸). Despite their cost advantage, Sn–Cu solders generally exhibit lower mechanical strength and reduced thermal fatigue resistance compared with Ag-containing alloys (Cheng *et al.*, 2017 ▸). Therefore, to improve the reliability of Sn–Cu solders, it is essential to investigate their microstructural evolution and deformation behavior, particularly under thermal cycling conditions.

In this paper, we apply *i*-S3DXRD to investigate the orientation evolution of β-Sn grains in two die-attach solder joints subjected to different thermal cycling conditions. With the 3D scan mode, we obtained a high-resolution three-dimensional orientation map of the β-Sn layer in a die-attach solder joint subjected to a low-temperature, short-duration thermal cycling condition (−65 °C for 15 min / +150 °C for 15 min). In contrast, with the point-by-point scan mode, we acquired a wide-area 2D orientation map of the β-Sn layer subjected to a high-temperature, long-duration thermal cycling condition (−40 °C for 30 min / +175 °C for 30 min). From these two measurements, we found that specific crystallographic axes of β-Sn grains tend to align with the normal direction (ND) of the Cu substrate. We attribute this reorientation to recrystallization-assisted grain boundary migration and/or grain coalescence during thermal cycling.

## 2. Methods

### 2.1. Principle of *i*-S3DXRD

**Figure 1:**
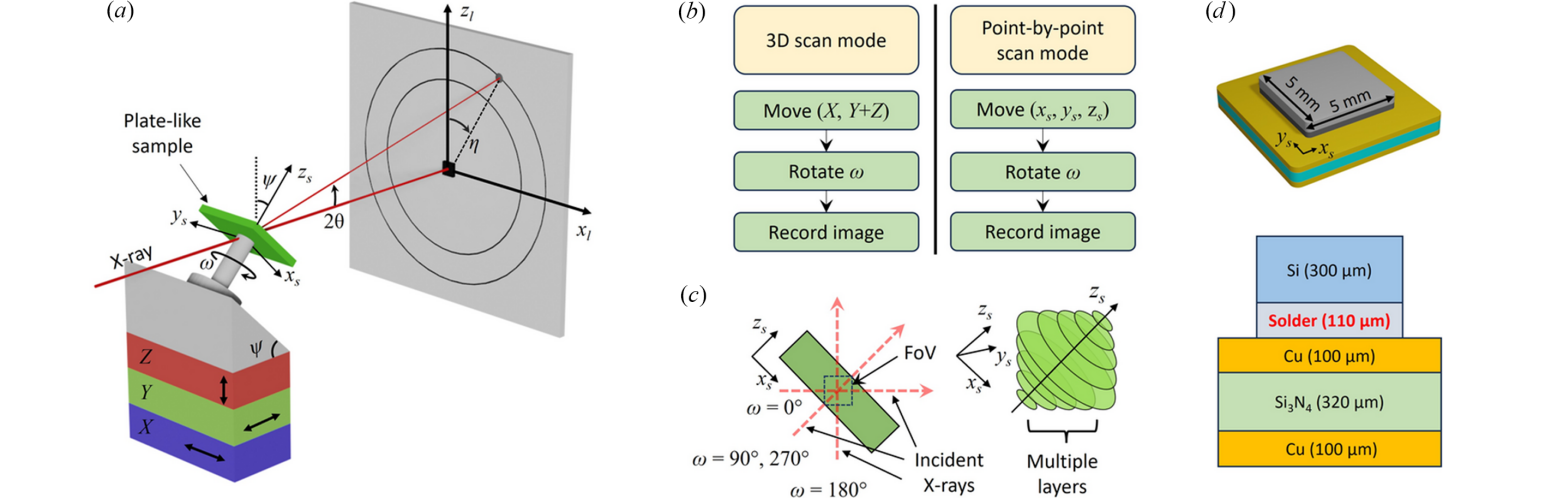
An illustration of the experimental configuration of *i*-S3DXRD. (*a*) A triangular-shaped aluminium block with an angle of Ψ is mounted on the (*X*, *Y*, *Z*)-translation stages. Then, a rotation stage (ω) is mounted on the triangular aluminium block. After mounting a sample holder transparent to X-rays, a Pb-free solder joint is mounted on top of the holder. (*b*) Two different scan modes are described. In 3D scan mode, (*X*, *Y*, *Z*) stages mounting the triangular block move first, the sample rotates, then the diffraction image is recorded. The sample rotation axis moves together with the (*X*, *Y*, *Z*) stages. In the case of a point-by-point scan mode, the sample stage (*x*_s_, *y*_s_, *z*_s_) moves first, then the sample rotates. Finally, capture the diffraction image. In this configuration, the rotation center does not move. (*c*) The field of view (FoV) of *i*-S3DXRD with 3D scan mode defined by the inclination angle and incident X-ray beam trajectories shows the attachment of the double cones sharing the base. (*d*) A 3D view and a cross-sectional view of the die-attach solder joint specimen are shown. The specimen consists of a solder layer mounted on a DBC substrate, which is covered by a silicon layer.

The experimental principle of *i*-S3DXRD is illustrated in Fig. 1 ▸(*a*). On top of (*X*, *Y*, *Z*)-translation stages, a metal block is mounted with the inclination angle of Ψ. After that, a rotation stage is mounted, which has the form of X-ray CL. By installing a Pb-free solder joint on the holder transparent to the incident X-rays, we can avoid the high X-ray absorption at any rotation angle, even for the plate-like sample. Note that the conventional S3DXRD is more suitable for the symmetric matchstick-like specimen to maintain the X-ray diffraction strong enough. See the supplementary material (Quantifying angular loss due to X-ray absorption in vertical rotation geometry) for additional details on X-ray absorption under the sample rotation geometry. The relationship between *i*-S3DXRD and S3DXRD is similar to that of X-ray CL and X-ray CT.

The two different scan modes of *i*-S3DXRD are described in Fig. 1 ▸(*b*). The first one is a 3D scan mode whose rotation axis moves during the scanning. The (*x*_s_, *y*_s_, *z*_s_) axes are fixed, while the (*X*, *Y*, *Z*) axes are moved to illuminate the grain in the field of view (FoV). The diffraction data set passing through a voxel (*x*_s_, *y*_s_, *z*_s_) can be selected after the whole scan. In this mode, we can select the fractional coordinate of the (*x*_s_, *y*_s_, *z*_s_), if we consider the X-ray beam trajectories and X-ray beam width. The voxel size defined by the translational step of the specimen can be subdivided into fractional numbers, which provide higher resolution than the point-by-point scan mode.

The second one is a point-by-point scan mode, which makes it easy to obtain the orientation of the selected voxel in a short time. In this mode, the sample rotates at a given sample coordinate (*x*_s_, *y*_s_, *z*_s_). Note that the (*X*, *Y*, *Z*) axes do not move in this mode. We can consider that the crystallite rotating at (*x*_s_, *y*_s_, *z*_s_) has the maximum number of diffraction peaks of β-Sn. By finding diffraction peaks and orientations over the multiple (*x*_s_, *y*_s_, *z*_s_), orientation maps are obtained. In this mode, one diffraction image set is needed for the orientation at a given (*x*_s_, *y*_s_, *z*_s_), which is suitable for the surveying of the wide area.

The point-by-point mode provides a large FoV with short acquisition time, making it suitable for surveying orientation changes over wide areas, albeit with lower spatial resolution. In contrast, the 3D scan mode yields high-resolution volumetric reconstructions with clearly defined grain boundaries, but requires substantially longer measurement time. Thus, the 3D scan captures detailed microstructural features, whereas the point-by-point mode enables efficient large-area coverage under time-limited experimental conditions.

The FoV of *i*-S3DXRD with the 3D scan mode is illustrated in Fig. 1 ▸(*c*). The accessible FoV can be obtained from the direct X-ray beam trajectories. A diffraction image set measured under ω = 0° to 360° passing through the sample coordinate (*x*_s_, *y*_s_, *z*_s_) is necessary for the orientation reconstruction of the selected voxel. The whole volume of the FoV to be allowed has the form of a double cone shape sharing the basement with super-resolution smaller than the incident X-ray beam size (Kim *et al.*, 2023*a* ▸; Kim *et al.*, 2023*b* ▸). The translation of the sample and Ψ constrain the direct X-ray beam trajectories in the sample coordinate, which determines the shape of the FoV. In this work, we set the Ψ and voxel size to 45° and 10 µm, respectively.

### 2.2. Sample preparation

We prepared two identical Pb-free die-attach solder joints between a Si chip and a directly bonded copper (DBC) substrate, for which the *i*-S3DXRD analysis was performed to investigate the recrystallization process of β-Sn before and after the thermal cycles. The β-Sn phase present in the solder joints has a body-centered tetragonal crystal structure (space group *I*4_1_/*amd*) with unit-cell parameters *a* = 5.83 Å and *c* = 3.18 Å. The first one was measured by the 3D scan mode for mapping with a high resolution, while the second one was measured by the point-by-point scan mode for analyzing a wide area. A schematic diagram of the die-attach solder joint specimen is illustrated in Fig. 1 ▸(*d*). A DBC substrate composed of Cu (100 µm) / Si_3_N_4_ (320 µm) / Cu (100 µm) with the dimension of 20 mm × 20 mm was prepared (Tatsumi *et al.*, 2024 ▸). On top of the DBC substrate, a Sn-0.75 mass% Cu (Sn-0.75Cu) solder sheet and a Si chip with the metallization layer of 0.7 nm Ni/0.1 nm Au were attached. The dimensions of the solder sheet and Si chip were 5 mm × 5 mm × 110 µm and 5 mm × 5 mm × 300 µm, respectively. The die-attach solder joint was fabricated by a reflow oven (SK-5000, Sanyoseiko Co., Ltd, Japan) under 240 °C for 60 s, followed by furnace cooling in an N_2_ atmosphere. The microstructure of the Sn-0.75Cu solder consists of β-Sn matrix and tiny dispersed Cu_6_Sn_5_ intermetallic compounds (IMCs) (Seo *et al.*, 2009 ▸). We chose two different temperature conditions to capture different thermal fatigue phenomena. The low-temperature, short-duration condition (−65°C for 15 min / +150 °C for 15 min) was selected to emphasize the effects of high thermal stress. In contrast, the high-temperature, long-duration condition (−40 °C for 30 min / +175°C for 30 min) was chosen to highlight the significant microstructural changes that are more pronounced at elevated temperatures over time. The thermal cycling was conducted under air environment using a chamber. The first die-attach solder joint was subjected to 250 thermal cycles (−65 °C for 15 min / +150 °C for 15 min). The second die-attach solder joint was subjected to 376 thermal cycles (−40 °C for 30 min / +175 °C for 30 min).

### 2.3. Experimental setup

The experiment was performed at BL29XU, the RIKEN Coherent X-ray Optics beamline, of SPring-8. An X-ray energy of 37 keV was selected by a Si (111) double crystal monochromator (DCM). After focusing the selected X-rays with a mirror, the beam was trimmed to be the size of 20 µm × 20 µm in the horizontal and vertical directions, respectively. After the installation of the (*X*, *Y*, *Z*) translation stage, an Al block inclined to the [011] direction (45° inclined to the +*Y*-direction) was mounted. A sample was mounted on the sample holder made of polymethyl methacrylate (PMMA) almost transparent to X-rays of 37 keV. Then the holder with the sample was mounted on the rotation stage. Then the specimen position was adjusted by motion stages with an optical camera.

A flat panel detector (XRD 4343 C T, Varex Imaging) was installed 40 cm downstream of the sample rotation center. A 2 × 2 binning mode with an effective pixel size of 300 µm × 300 µm was set to reduce the measurement time since the frame rate of the detector depends on the pixel resolution. The X-ray exposure time was 150 ms, and the rotation speed was maintained at 20° s^−1^, corresponding to an angular sampling of Δω = 20° s^−1^ × 0.15 s = 3°.

The detector was externally triggered by the motor controller (PM16C-HW2) for every acquisition. Upon receiving the first trigger, the detector starts the exposure, and upon receiving the next trigger it completes the readout and immediately begins the next exposure. The dead time between exposures is on the order of 1 ms, which is negligible compared with both the 150 ms exposure and the 3° angular interval. Therefore, no measurable angular gap exists between successive frames, and the probability of missing diffraction peaks due to detector readout is negligible. Under the constant rotation speed, diffraction signals were integrated over angular intervals of 3°, and 120 images were generated by one rotation. By synchronizing the initial phase of the rotation and data acquisition time, the integrity of the data was kept.

The detector geometry (sample to detector distance, detector center, detector tilt) was calibrated using diffraction patterns from β-Sn (Borbely *et al.*, 2014 ▸). The calibrated 2θ–η map is shown in Fig. S3. The straightening of the diffraction rings confirms that the detector-tilt correction was successfully applied. The calibrated parameters were used consistently for subsequent reconstructions.

For 3D scan mode, the *X* range was ±1.28 mm with a 40 µm step, and to keep the same detector to rotation center distance we set a new virtual translation axis *S* composed of *Y* and *Z* stages with an inclination angle of 45° moving simultaneously with the same amount. The travel length of *S* was ±1.28 mm with a 40 µm step, the same as the *X* axis. After the scan, the first die-attach solder joint sample was detached from the holder, and the 250 thermal cycles (−65°C for 15 min / +150°C for 15 min) were applied. The thermal fatigued sample was mounted on the sample holder again under the same conditions, and the measurement was implemented with the same scan conditions as the pristine specimen.

The measurement ranges of each *x*_s_, *y*_s_ for the point-by-point scan mode were ±2 mm with a 100 µm step. After the scan, the specimen was detached from the holder, and the 376 thermal cycles (−40°C for 30 min / +175°C for 30 min) were applied. After the thermal cycling, the sample was mounted on the sample holder again, and then the measurement was implemented with the same scan conditions.

A full 3D *i*-S3DXRD scan required approximately 17 h. Because both fresh and thermally cycled states were measured, the total 3D acquisition time was ∼34 h. For 2D *i*-S3DXRD measurements, each scan required 2.5 h, resulting in ∼5 h for the two states (before/after thermal cycling). Representative diffraction images can be found in Fig. S1.

### 2.4. Multigrain indexing procedure

To determine the orientation of the selected voxel (*x*_s_, *y*_s_, *z*_s_), data associated with the position (*x*_s_, *y*_s_, *z*_s_) must be found. Since we are considering the inclination to the [011] direction, the inclination operation **Ψ** and the rotation operation **Ω** can be described by
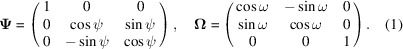
Here, ψ is the inclination angle and ω is the sample rotation angle. In this work, we set the ψ to 45°; therefore, the overall rotation operation (Kim *et al.*, 2023*b* ▸) is given by
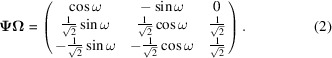
From this ***ΨΩ***, every rotated position of the selected voxel (*x*_s_, *y*_s_, *z*_s_) can be found. In the measurement procedure, we scanned by translating *X* and *S* stages under rotation. Therefore, we can find an *X*, *S*, and ω set, where each ω in the set is different. This calculation can be done over the voxel whole FoV, and the voxel size can be reduced according to a recent report that shows super-resolution smaller than the probe size (Kim *et al.*, 2023*a* ▸; Kim *et al.*, 2023*b* ▸).

After the image selection process, peak positions can be extracted by applying the threshold to each image. In some cases, extracted peaks are distributed over multiple images. Therefore, it is necessary to consider the connection of peaks over the rotation angle ω and to find the center of mass (COM) position of each peak. If this process is omitted, the indexing process will consider them as multiple peaks with different angular positions. We solved this problem by introducing a label-equivalent-resolving algorithm (He *et al.*, 2011 ▸; Kim *et al.*, 2026 ▸). By labeling the connected components in the 3D volume image, we found the COMs and converted them into (2θ, η) for the calculation of the **G**-vectors.

It is known that the **G**-vector is obtained from the **Ψ**, **Ω**, 3D orientation matrix **U**, and **B** matrix as shown below. The obtained value has the following relation with diffraction peak angles (2θ, η),
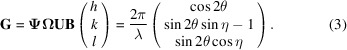
The **B** matrix includes reciprocal lattice constants (a*, b*, c*, α*, β*, γ*) and has the form
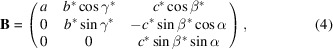
where the lattice constant α in the **B** matrix in real space is given by
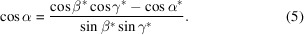
The obtained **G**-vector can be compared with the theoretical model to find **U**. The main goal of the multigrain indexing is to find the **U** which rotates the ideal (*h k l*)^T^ composed of an integer value. Therefore, if we find the **U**, we can transform the integer (*h k l*)^T^ into the obtained **G** vector.

**Figure 2:**
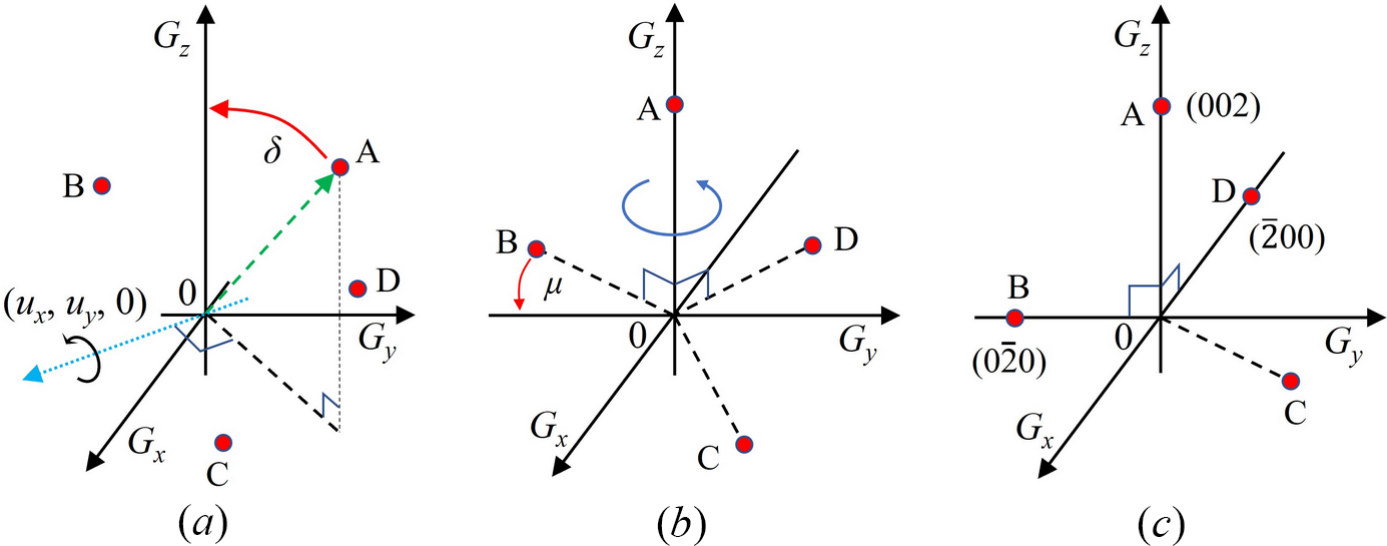
Schematic view of the multigrain indexing procedure. (*a*) A rotation operation along the (*u_x_*, *u_y_*, 0) axis by δ is applied to **G**-vectors so that an arbitrarily selected peak A is aligned to the *G_z_* axis. (*b*) **G** vectors are rotated by μ to align the peak B to the *G_y_* axis. (*c*) The overall rotation results show peaks A, B, and D are aligned with each axis, which indicates they are from the same voxel.

One simple way to find **U** is to compare the position of **G** vectors with a reference. Since we know ***ΨΩ***, we can obtain (***ΨΩ***)^−1^**G**, which is used for **UB** calculation. For example, we can set a reference orientation matrix **U** as an identity matrix, and a **B** matrix with ideal lattice constants. The next step is to select an arbitrary diffraction peak A in the same {*h k l*} family as illustrated in Fig. 2 ▸(*a*). After that, we can align the peak A to the *G_z_* axis and obtain a rotation matrix. The rotation angle δ is computed from the inner product of the peak A’s vector representation and unit vector to the *G_z_* axis. In this condition, the rotation axis is given by (*u_x_*, *u_y_*, 0), which is perpendicular to the *G_x_*, and *G_y_* components of peak A. The rotation around the axis can be described by the Rodrigues rotation matrix as follows,
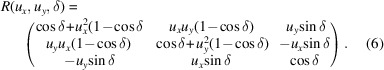
After the rotation operation to all peaks (A, B, C, and D), peak A will be aligned to the *G_z_* axis; however, other peaks will not be aligned to other axes. If we assume that A is aligned to (002), other peaks belonging to the same voxel should be (020), (200), (200), (200), and (002), since we selected the {002} family. Therefore, by calculating the angle μ between the *G_y_* axis and peaks in the (*G_x_*, *G_y_*) plane shown in Fig. 2 ▸(*b*), we can perform another rotation operation along the *G_z_* axis from the following,
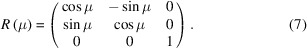
This rotation operation along the *G_z_* axis can be attempted on peaks B, C, and D. After this rotation operation, we find that peaks A, B, and D are aligned to the *G_x_*, *G_y_*, and *G_z_* axes, and these three peaks can be indexed into (002), (200), and (200) as described in Fig. 2 ▸(*c*). In this situation, we can infer that peak C is not from the same voxel since the peak is away from the reference position.

The overall rotation operation to the peaks is**R**(μ)**R**(*u_x_*, *u_y_*, δ), and we obtain **U**^−1^. Because the observed diffraction peak positions can deviate from the ideal positions, if we multiply (**UB**)^−1^ by (***ΨΩ***)^−1^**G**, then (*h k l*)^T^ is slightly different from the integer value. However, in principle, the peak index should be an integer, and, therefore, after changing (*h k l*)^T^ into the nearest integer we obtain an integer index (*h*′*k*′*l*′)^T^. From **UB**(*h k l*)^T^(*h*′*k*′*l*′)^−T^, we obtain a new **UB** matrix having an integer index. From the orthogonal property of the rotation matrix given by **U**^−1^ = **U**^T^, the **BB**^T^ matrix can be obtained from the following relation,
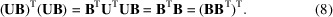
From the Cholesky decomposition, we obtain the **B** matrix. Then, we obtain lattice constants from the computed **B** matrix. This **B** matrix is slightly deviated from the ideal value, which gives the real lattice constant of the selected voxel. By multiplying **B**^−1^ by the right side of **UB**, we obtain the **U** matrix of the given voxel. From the multiplication of (**UB**)^−1^ to all diffraction peaks, we obtain the indexed diffraction peak positions and the number of indexed peaks belonging to the voxel.

Since there may be more than one **U** and **B** candidate, it is necessary to find the most probable **U** and **B**. This can be found by normalizing *N* (the number of indexed peaks) with *M* (the theoretical number of peaks under the given orientation). From *N*/*M*, the most probable orientation **U** of the selected voxel can be chosen. The overall procedure can be carried out for all available voxels, and we obtain a 3D orientation map.

For the reconstruction of the orientation map, we developed a custom Python code (currently not publicly released) that is fully parallelized and designed for execution on a distributed cluster. Each worker node first loads its assigned diffraction images and converts them into sparse matrices after applying an intensity threshold. These sparse images are transferred to the master node, which assembles them into a single dataset. Then the master node computes the image list required for each voxel. The voxel list is then distributed across the compute nodes, and each node further parallelizes its assigned voxels across its CPU cores. After multigrain indexing, each worker node returns the results to the master node, which assembles the complete 3D volume.

In this work, we initially reconstructed orientations for all 261 layers and then identified the β-Sn layer using the completeness map. The central layers of the fresh and thermally cycled volumes were aligned by matching grain positions. For 3D visualization and grain segmentation, we selected ten consecutive β-Sn layers. Grain extraction was performed using a 1° misorientation threshold, and each grain was represented as a set of voxels in 3D space. For visualization, voxelized grains were converted into polygonal surfaces as follows: (i) interior voxels were removed to reduce mesh size; (ii) surface voxels were converted into triangular meshes; and (iii) individual triangular faces were colored according to the IPF. Mesh generation was performed using the Python *Blender* module bpy (https://download.blender.org/pypi/bpy/). The final 3D rendering was performed in *Blender* 3.6 after importing all reconstructed grain meshes.

The computing cluster used in this work consisted of 11 worker nodes and one master node. Each worker node was equipped with dual-socket Intel Xeon Gold 6248R processors and 196 GB of RAM, with all nodes interconnected via Mellanox InfiniBand (56 Gbps). The storage system comprised twenty-four 4 TB SATA SSDs configured in RAID-10 to provide both high throughput and data redundancy. The typical processing time after measurement was approximately two hours. The initial stage—loading the diffraction images and converting them into sparse matrices—required about 30 min, depending on the complexity of the diffraction patterns. The full reconstruction of ∼2 million voxels required approximately 90 min. Because the diffraction patterns of the die-attach solder joint are relatively simple, the processing time is shorter than for more complex metallic systems. When the reconstruction parameters are known in advance, the pure computation time is generally below 2 h.

## 3. Results

### 3.1. 3D scan results for the first specimen

**Figure 3:**
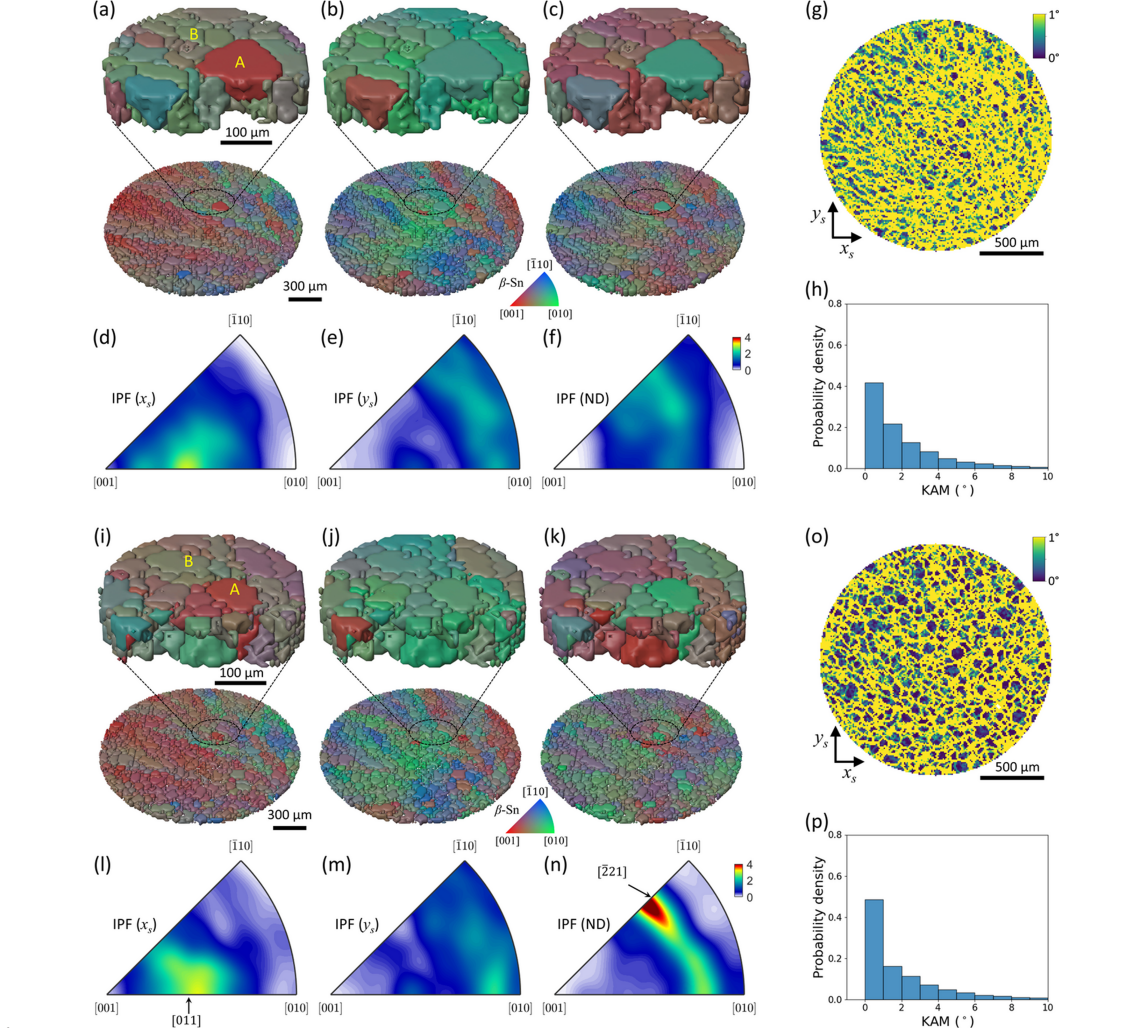
IPF maps, IPF density, grain boundary map, and kernel average misorientation map of the first specimen reconstructed from the 3D scan mode before and after thermal cycling. (*a*) The IPF (*x*_s_) map, (*b*) IPF (*y*_s_) map, and (*c*) IPF (ND) map of the first specimen with their high-magnifications before thermal cycling. Their IPF densities of the (*d*) *x*_s_ axis, (*e*) *y*_s_ axis, and (*f*) ND are described. The KAM map calculated with a 1° cut-off angle (*g*) and its statistical distribution before thermal cycling (*h*) are presented. The 3D IPF maps of the (*i*) *x*_s_ axis, (*j*) *y*_s_ axis, and (*k*) ND after thermal cycling show noticeable changes in orientation. Their IPF densities of the (*l*) *x*_s_ axis, (*m*) *y*_s_ axis, and (*n*) ND are shown. The KAM map after thermal cycling (*o*) and its statistical distribution (*p*) are also displayed. For individual grains shown in (*a*) and (*i*), grain A is broken into multiple small grains, while grain B becomes larger.

The reconstructed 3D IPF maps and IPF density map of β-Sn in the first specimen before thermal cycling are displayed in Fig. 3 ▸. The 10 µm voxel size, four times smaller than the scan step, was achieved by the super-resolution technique using the concept of the sub-voxel by tracing the incident X-ray beam trajectories in the sample coordinates (Kim *et al.*, 2023*a* ▸; Kim *et al.*, 2023*b* ▸). The orientation of each pixel is expressed by the color key of the IPF in the *x*_s_, *y*_s_, and ND. For example, the red color ([001] corner of the IPF color key) in IPF (ND) indicates that the *c* axis of β-Sn is parallel to the ND direction, and the green color ([010] corner of the IPF color key) means that the *a* axis of β-Sn is parallel to the ND. A gradual change in the color indicates a gradual change in the orientation of the grain due to a low-angle grain boundary (LAGB). In contrast, an abrupt change in the color implies the existence of the high-angle grain boundary (HAGB).

High-magnification IPF (*x*_s_, *y*_s_, ND) maps of the center before thermal cycling are shown in Figs. 3 ▸(*a*)–3 ▸(*c*), 3 ▸(*i*)–3 ▸(*k*), and Videos S1 and S2. To achieve a high sensitivity to the orientation difference, we deliberately set the misorientation threshold as low as 1° (see Fig. S2 in the supplementary material for more information). Small-sized grains with LAGB are continuously arranged in the parent grain, which forms a stripe pattern in a diagonal direction. Some single grains buried in the parent grain show abrupt changes in their orientation, which may be related to the inhomogeneous soldering process. In the case of grain A in Figs. 3 ▸(*a*) and 3 ▸(*i*), it is broken into multiple small grains after thermal cycling. Unlike grain A, grain B in Figs. 3 ▸(*a*) and 3 ▸(*i*) shows grain growth after thermal cycling. Initially, its neighboring grains have similar crystallographic orientations, which makes it easy to form a large single grain by thermal energy.

Figs. 3 ▸(*d*), 3 ▸(*e*), and 3 ▸(*f*) show the IPF (*x*_s_), IPF (*y*_s_), and IPF (ND) density of the pristine β-Sn of the first sample over the specimen evaluated from Figs. 3 ▸(*a*), 3 ▸(*b*), and 3 ▸(*c*), respectively. Fig. S5 presents a scatter plot of IPFs over the specimen. The IPF densities to the *x*_s_ axis, *y*_s_ axis, and ND after thermal cycling of the first sample evaluated from Figs. 3 ▸(*i*), 3 ▸(*j*), and 3 ▸(*k*) are illustrated in Figs. 3 ▸(*l*), 3 ▸(*m*), and 3 ▸(*n*), respectively. In the case of IPF (*x*_s_, *y*_s_) density, there are no significant changes in texture after thermal cycling. In contrast, IPF (ND) density shows an enhancement around [211] after thermal cycling. Two-dimensional orientation maps of additional layers before and after thermal cycling are provided in the supplementary material (Figs. S7–S16).

To evaluate the intragranular misorientation, the kernel average misorientation (KAM) map was calculated. The KAM at voxel (*i*, *j*, *k*) is calculated as the average mis­orientation between that voxel and its six first-nearest neighbors, evaluated within the three-dimensional volumetric orientation dataset. Let *w*(*o*_1_, *o*_2_) denote the misorientation angle between orientations *o*_1_ and *o*_2_. Then, the KAM for the first-neighbor configuration is defined as follows,
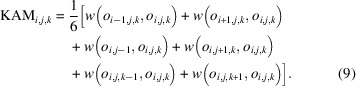
The KAM maps calculated before and after the thermal cycling test are shown in Figs. 3 ▸(*g*) and 3 ▸(*o*), respectively. To emphasize intragranular misorientation, a cut-off angle of 1° was applied. After thermal cycling, regions with KAM values close to 0° tend to follow the grain boundary morphology, indicating an improvement in the crystallinity within the grains. The statistical distributions of KAM before and after thermal cycling, evaluated from voxels with KAM, are shown in Figs. 3 ▸(*h*) and 3 ▸(*p*), respectively. The histograms clearly indicate a systematic shift of the KAM distribution toward lower misorientation angles after thermal cycling. This observation is similar to the recovery behavior of deformed aluminium during annealing observed by DAXM (Yu *et al.*, 2023 ▸). They observed a shift of the misorientation distribution toward lower angles after annealing and attributed this behavior to subgrain boundary migration and subgrain coalescence.

**Figure 4:**
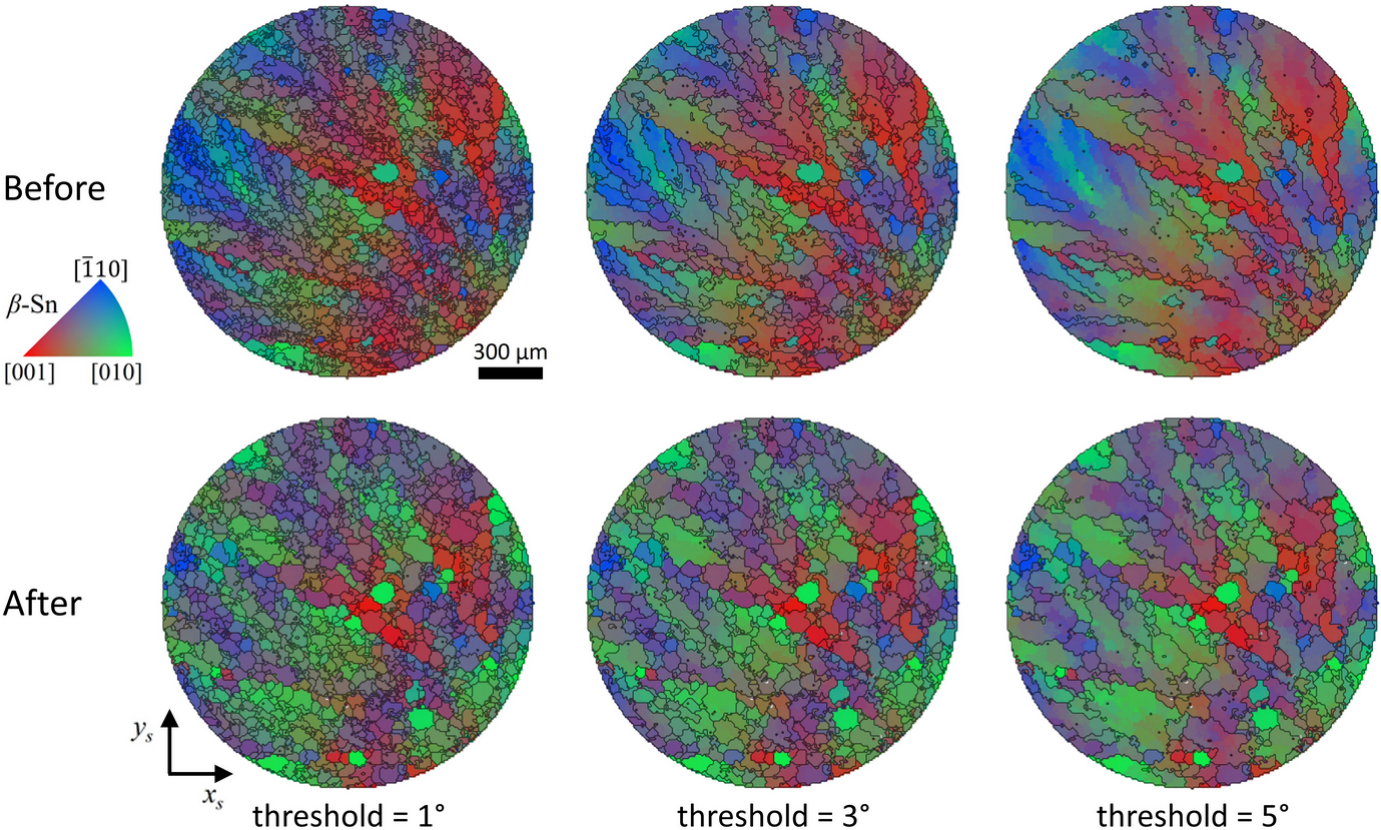
Overlays of IPF (ND) maps and grain boundaries before and after thermal cycling, shown with different misorientation angle thresholds (1°, 3°, and 5°). Before thermal cycling, low-threshold (1°) maps reveal numerous small grains, while higher thresholds (3° and 5°) show the formation and merging of multiple grains. After thermal cycling, grain growth is observed at the 1° threshold, and grains become more rounded. At the 3° and 5° threshold, grain fragmentation and boundary rounding are shown.

Overlays of IPF (ND) maps and grain boundaries with various misorientation angle thresholds of 1°, 3°, and 5° are shown in Fig. 4 ▸. Before thermal cycling, grain boundaries defined using a 1° threshold reveal numerous small grains. When the threshold is increased to 3°, the grains exhibit elongated and anisotropic branches. At a 5° threshold, these branches tend to merge into larger grains, although some HAGBs remain distinct and unmerged. After thermal cycling, the grain boundaries defined by a 1° threshold show slightly larger grains than in the pristine state, indicating modest grain growth. At the 3° threshold, neighboring grains merge into larger grains, but the branch features become less distinct. At the 5° threshold, elongated grains with rounded boundaries appear, and the overall grain size becomes smaller than in the pristine specimen.

From the perspective of low misorientation thresholds, the β-Sn grains undergo grain growth driven by thermal energy through grain boundary migration or grain coalescence. As a result, branch structures become more rounded and smoother after thermal cycling. Conversely, from the perspective of high misorientation thresholds, large parent grains appear fragmented into smaller grains, reflecting microstructural degradation associated with thermal fatigue.

**Figure 5:**
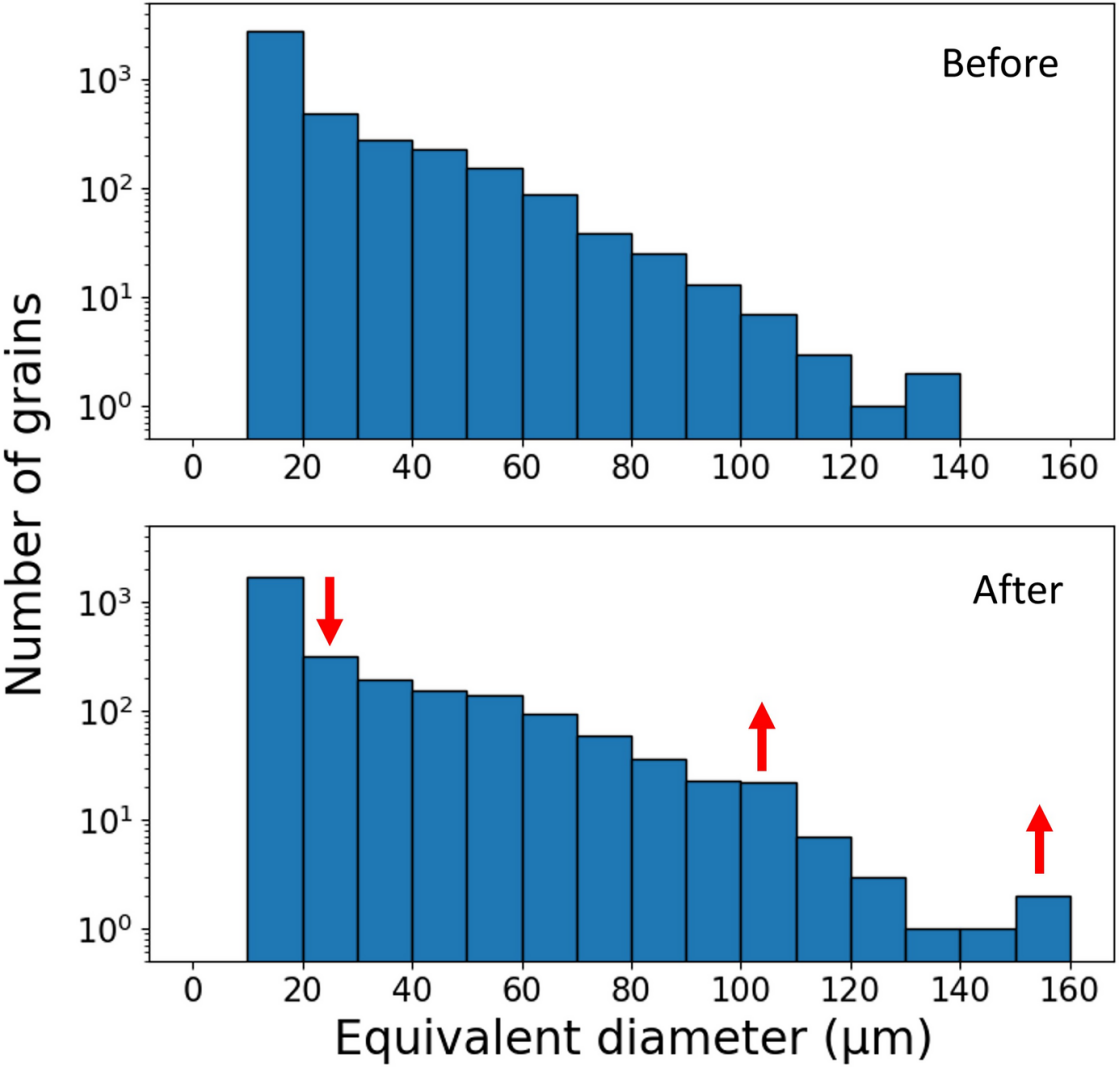
Histograms of grain size distribution before and after thermal cycling, evaluated using a 1° misorientation threshold. Recrystallization and grain growth during thermal cycling result in the formation of larger grains with a simplified grain boundary network. The number of small grains with diameters near 20 µm decreases, while the population of grains larger than 100 µm increases significantly after thermal cycling.

For a more detailed statistical analysis, we calculated the equivalent circle diameter of individual grains using a 1° misorientation threshold. Fig. 5 ▸ shows the grain size distributions before and after thermal cycling. The number of grains around 20 µm decreases, while the number of grains larger than 100 µm increases. Additionally, the largest grain size increases from 140 µm to 160 µm, suggesting the occurrence of grain growth during recrystallization and/or subsequent grain growth after recrystallization (Yu *et al.*, 2023 ▸).

The orientation variation was quantified by calculating the voxel-wise misorientation angle before and after thermal cycling. For this voxel-wise analysis, the central layer of the three-dimensional orientation map was selected based on the completeness map, where the completeness factor is defined as the ratio of the number of detected diffraction peaks to the theoretical number of peaks. Invariant points, corresponding to grains whose shapes remain nearly unchanged, were then identified between the corresponding two-dimensional slices. To minimize alignment errors, the voxel positions were systematically shifted, and the misorientation map and its statistical distribution were evaluated to identify the alignment that yields the lowest peak position and the narrowest distribution in the misorientation histogram. Although a tentative optimum alignment was obtained using this method, reconstruction-related errors may still affect the results. In particular, the intensity threshold used for diffraction-peak detection can influence the multi-grain indexing. Because diffraction intensities may change due to heating during the thermal cycling test, the choice and optimization of the intensity threshold can consequently affect the resulting misorientation distribution.

**Figure 6:**
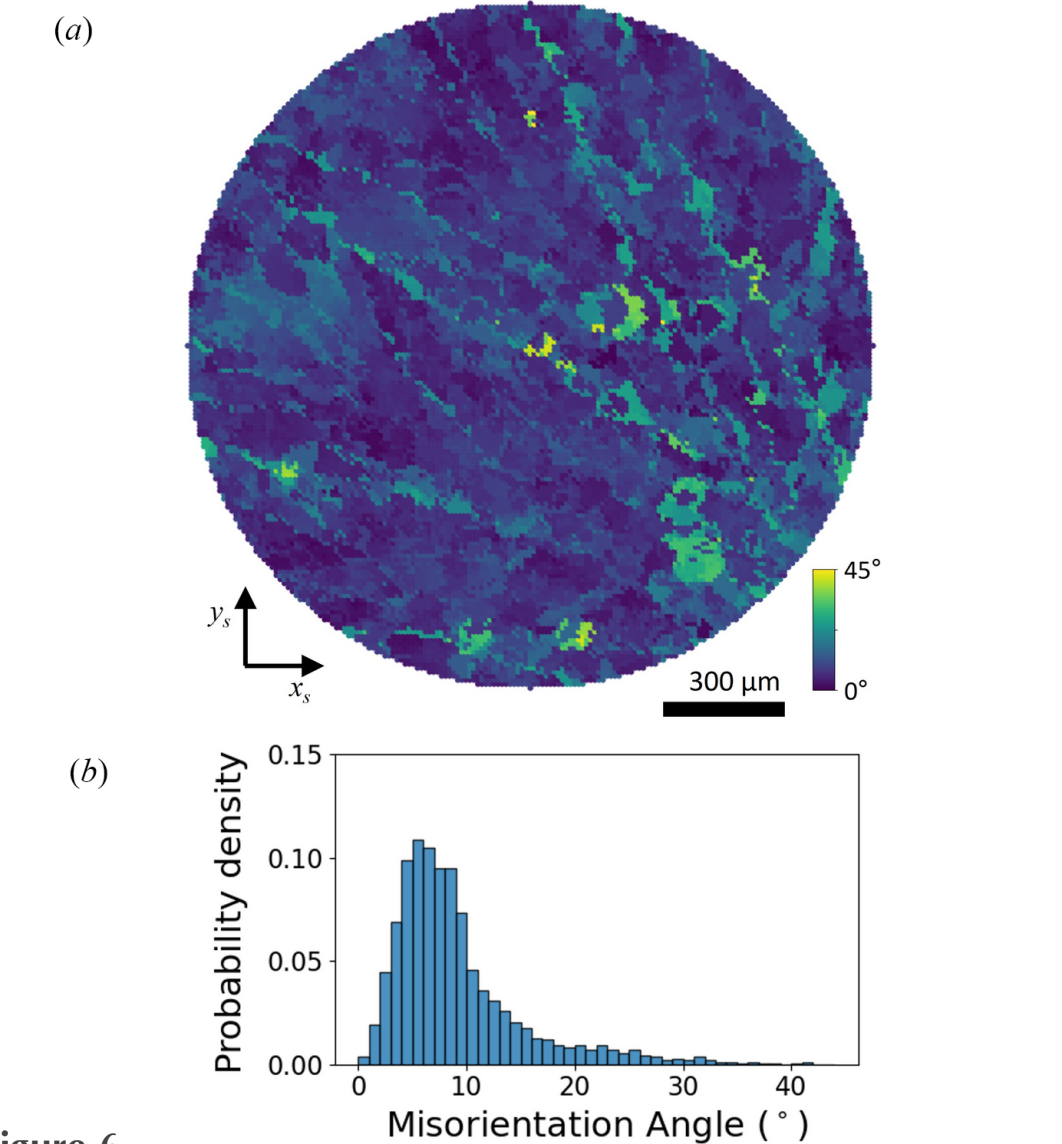
Orientation variation by thermal cycling test. (*a*) Orientation-variation map showing inhomogeneous local rotation across the observed region, with branch-like features resembling the grain-boundary morphology of the fresh state. (*b*) Histogram of voxel-wise misorientation angles, exhibiting a peak near 6°.

The resulting 2D orientation-variation map in Fig. 6 ▸(*a*) reveals misorientation across the observed region, with branch-like features that resemble the grain-boundary morphology of the fresh state. The misorientation spans a broad range from 0° to 45°. The corresponding histogram in Fig. 6 ▸(*b*) exhibits a pronounced peak near 6°, indicating the average reorientation angle induced by thermal cycling. On the other hand, large misorientation angles, although their threshold is not entirely clear, such as those exceeding 30°, are predominantly associated with grain boundary migration. Due to the grain boundary migration, extended voxels and pristine voxels at the same specimen coordinates may belong to two distinct grains. Therefore, the misorientation angle is calculated between different grains, which broadens the mis­orientation distribution.

**Figure 7:**
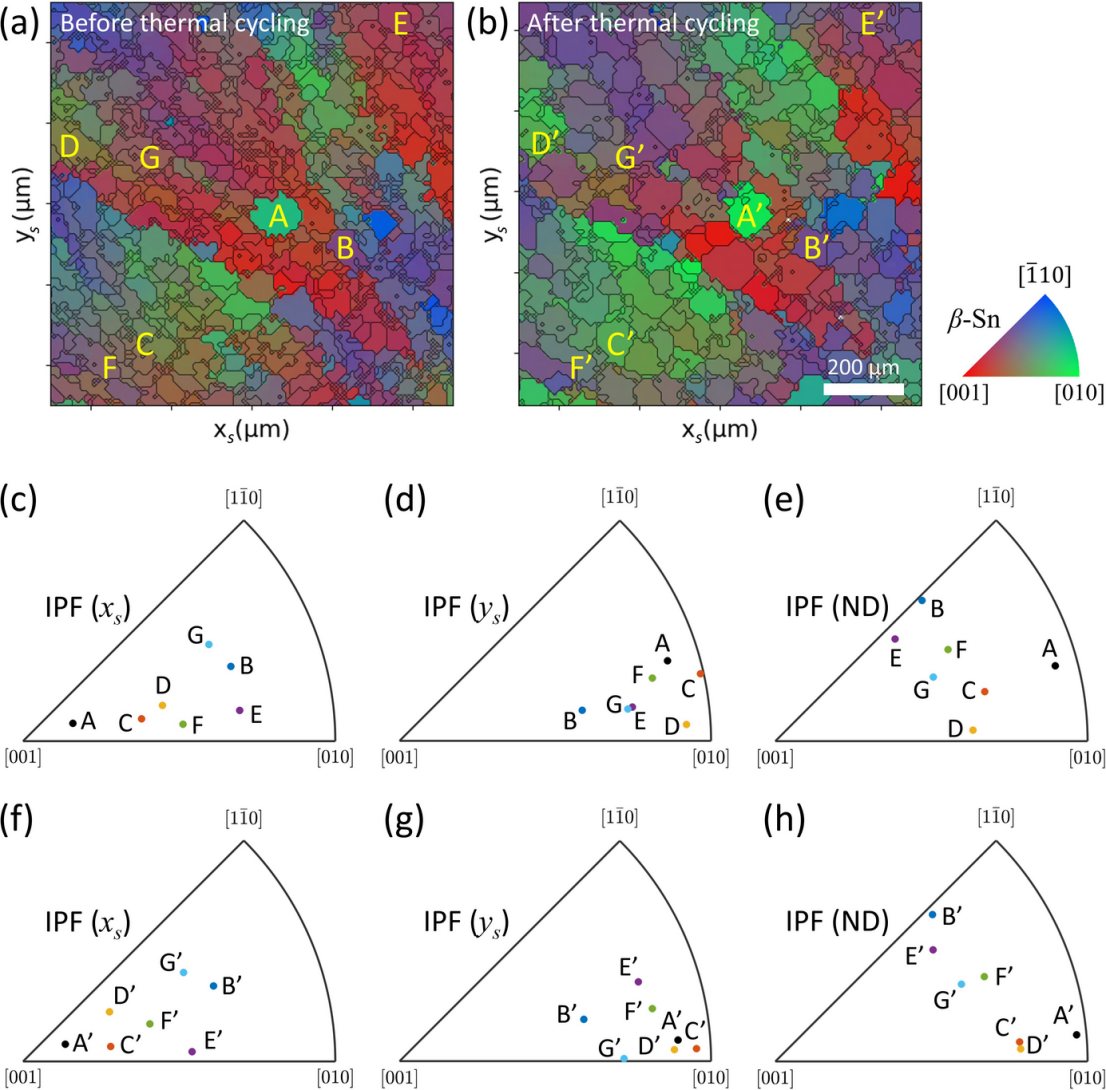
Reorientation of individual grains by thermal cycling test. IPF (ND) maps of the β-Sn solder in the first specimen are shown before (*a*) and after (*b*) thermal cycling. The orientations of selected grains (A, B, C, D, E, F, and G) before thermal cycling are presented in IPFs (*c*, *d*, and *e*), while the orientations of the same grains (A′, B′, C′, D′, E′, F′, and G′) after thermal cycling are shown in IPFs (*f*, *g*, and *h*).

To understand the reorientation of individual grains, we traced the orientation of selected grains using IPF maps. No post-processing was employed on the orientation maps. The selected grains (A, B, C, D, E, F, and G) in their pristine state are shown in the IPF (ND) map in Fig. 7 ▸(*a*). Their orientations in the IPF (*x*_s_), IPF (*y*_s_), and IPF (ND) are presented in Figs. 7 ▸(*c*), 7 ▸(*d*), and 7 ▸(*e*), respectively. After the thermal cycling test, the same grains (A′, B′, C′, D′, E′, F′, and G′) are reoriented, as shown in Fig. 7 ▸(*b*). Their post-thermal-cycling orientations in the IPF (*x*_s_), IPF (*y*_s_), and IPF (ND) are illustrated in Figs. 7 ▸(*f*), 7 ▸(*g*), and 7 ▸(*h*), respectively.

For grain A, the *a* axis rotated toward the normal direction (ND). In contrast, grain B remained nearly static during thermal cycling. Grains C and D exhibited reorientation behavior similar to that of grain A. For grain E, the *c* axis rotated toward the specimen *x*_s_ axis. In the case of grain F, the *a* axis rotated slightly toward the specimen ND, while the *c* axis rotated slightly toward the specimen *x*_s_ axis. For grain G, the *c* axis slightly rotated toward the specimen *x*_s_ axis, and a direction intermediate between the [001] and [010] crystal directions was aligned with the specimen *y*_s_ axis, which is distinct from other grains.

To validate the accuracy of *i*-S3DXRD and the associated analysis, it is necessary to confirm the results using an independent method such as EBSD, which is a standard technique for crystallographic orientation mapping. Although *i*-S3DXRD has previously been demonstrated on metal wire specimens, its results have not yet been directly validated against EBSD. To address this, EBSD measurements were conducted on the first sample after the thermal cycling test, following completion of the *i*-S3DXRD measurements.

For the EBSD measurements, the silicon layer above the solder was polished to expose a clean surface suitable for EBSD analysis. The EBSD scan was performed with a step size of 12.5 µm using a hexagonal grid, covering a total area of approximately 5.5 mm × 5.5 mm. After acquisition, the corresponding measurement positions were identified, and the hexagonal EBSD grid was converted to a square grid to enable direct comparison with the *i*-S3DXRD data. For each voxel in the square grid, the crystallographic orientation was assigned from the nearest EBSD voxel without interpolation, in order to preserve the original orientation information. No post-processing was applied to both *i*-S3DXRD and EBSD.

**Figure 8:**
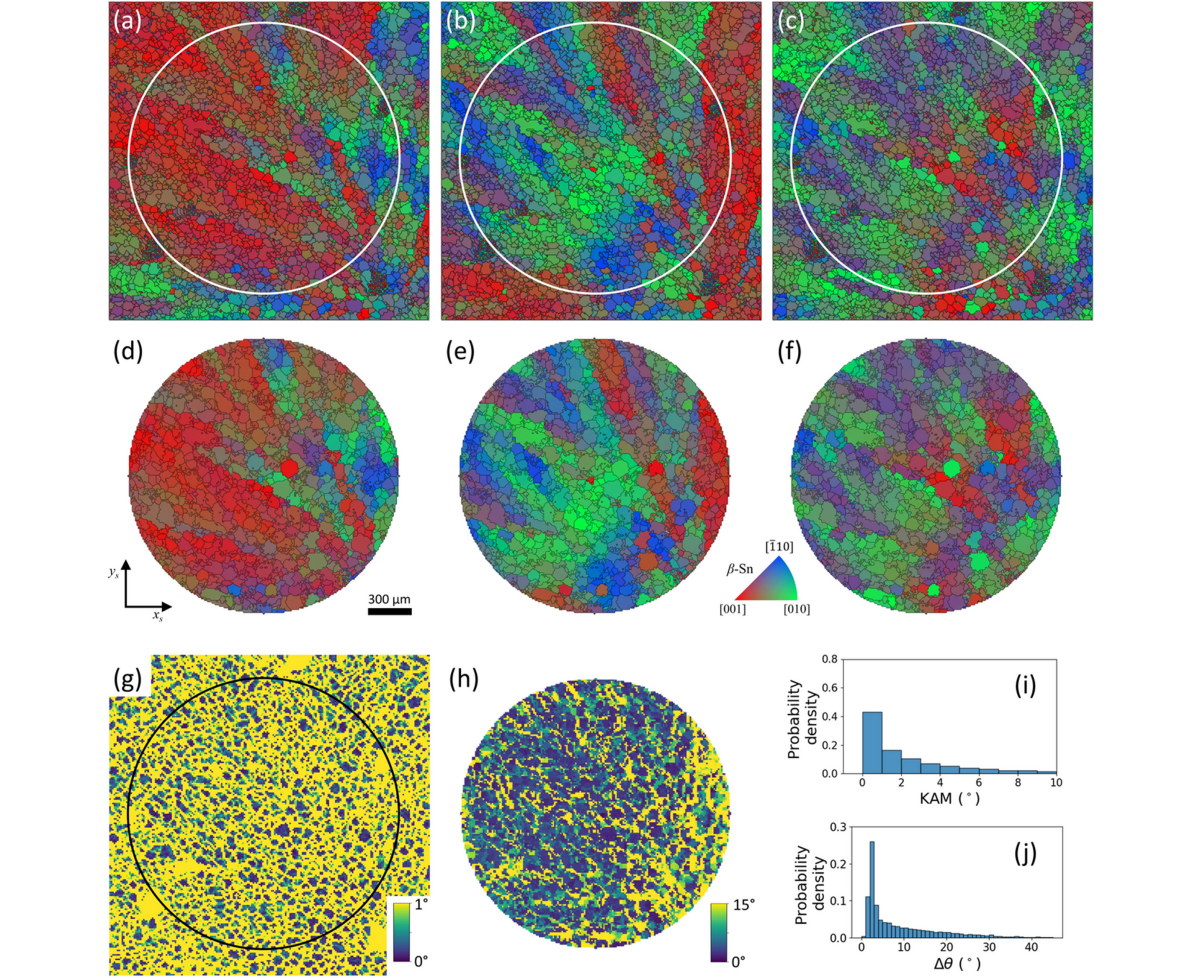
Comparison of orientation maps obtained by *i*-S3DXRD and EBSD for the solder sample after 250 thermal cycles. (*a*) IPF (*x*_s_) map, (*b*) IPF (*y*_s_) map, and (*c*) IPF (ND) map of the first specimen after 250 thermal cycles, measured by EBSD. (*d*) IPF (*x*_s_) map, (*e*) IPF (*y*_s_) map, and (*f*) IPF (ND) map of the same specimen measured by *i*-S3DXRD. (*g*) KAM map calculated from the EBSD data using a cut-off angle of 1°. (*h*) Angular deviation map evaluated by comparing the EBSD and *i*-S3DXRD results. (*i*) Histogram of KAM values obtained from the EBSD map. (*j*) Statistical distribution of angular deviation derived from (*h*).

The IPF maps along the *x*_s_, *y*_s_, and ND directions obtained from EBSD are shown in Figs. 8 ▸(*a*), 8 ▸(*b*), and 8 ▸(*c*), respectively, together with grain boundaries defined using a mis­orientation threshold of 1°. The corresponding IPF maps from the same layer obtained by *i*-S3DXRD are presented in Figs. 8 ▸(*d*)–8 ▸(*f*). The circular outlines in Figs. 8 ▸(*a*)–8 ▸(*c*) indicate the identical area in Figs. 8 ▸(*d*)–8 ▸(*f*). The two datasets exhibit similar microstructural features in all IPF maps. However, the grain boundaries in the EBSD maps appear more intricate despite the use of the same misorientation threshold. In addition, grain shapes in the EBSD maps appear sharper, whereas those obtained by*i*-S3DXRD are comparatively smoother and more rounded.

The KAM was calculated from the EBSD data to confirm the consistency of the intragranular misorientation, as shown in Fig. 8 ▸(*g*). The same calculation algorithm and color scale were applied to enable direct comparison with the *i*-S3DXRD results. Clusters of low KAM values are observed, delineating the grain morphology similar to Fig. 3 ▸(*o*). The corresponding KAM histogram is shown in Fig. 8 ▸(*i*), exhibiting a maximum below 1° and a gradual decrease toward higher misorientation angles. This trend is consistent with that observed in Fig. 3 ▸(*p*).

To compare the angular consistency of the two methods, the two orientation maps were directly overlaid, and the mis­orientation angle (Δθ) between corresponding voxels was calculated. The alignment procedure used for this analysis is identical to that applied in Fig. 6 ▸. As shown in Fig. 8 ▸(*h*), low Δθ values are observed near the grain centers, whereas larger Δθ values appear near the grain boundaries. Δθ exhibits a systematic variation along the *x*_s_ direction. To statistically characterize Δθ, the corresponding histogram is shown in Fig. 8 ▸(*j*). The distribution exhibits a maximum around 3°, decreases rapidly up to approximately 5°, and then gradually decays, extending to about 45°.

These differences may arise from the relatively large rotation step, sample translation step, and X-ray beam size used in the *i*-S3DXRD measurements. Therefore, a finer rotation step, a finer translation step, and a smaller beam size may be required to improve the reconstruction quality. In addition to measurement parameters, uncertainties in the multi-grain indexing process—such as intensity thresholds and *HKL* tolerance settings—may also influence the indexing results. Consequently, further optimization of the data-analysis and indexing algorithms is necessary.

### 3.2. Point-by-point scan results for the second specimen

**Figure 9:**
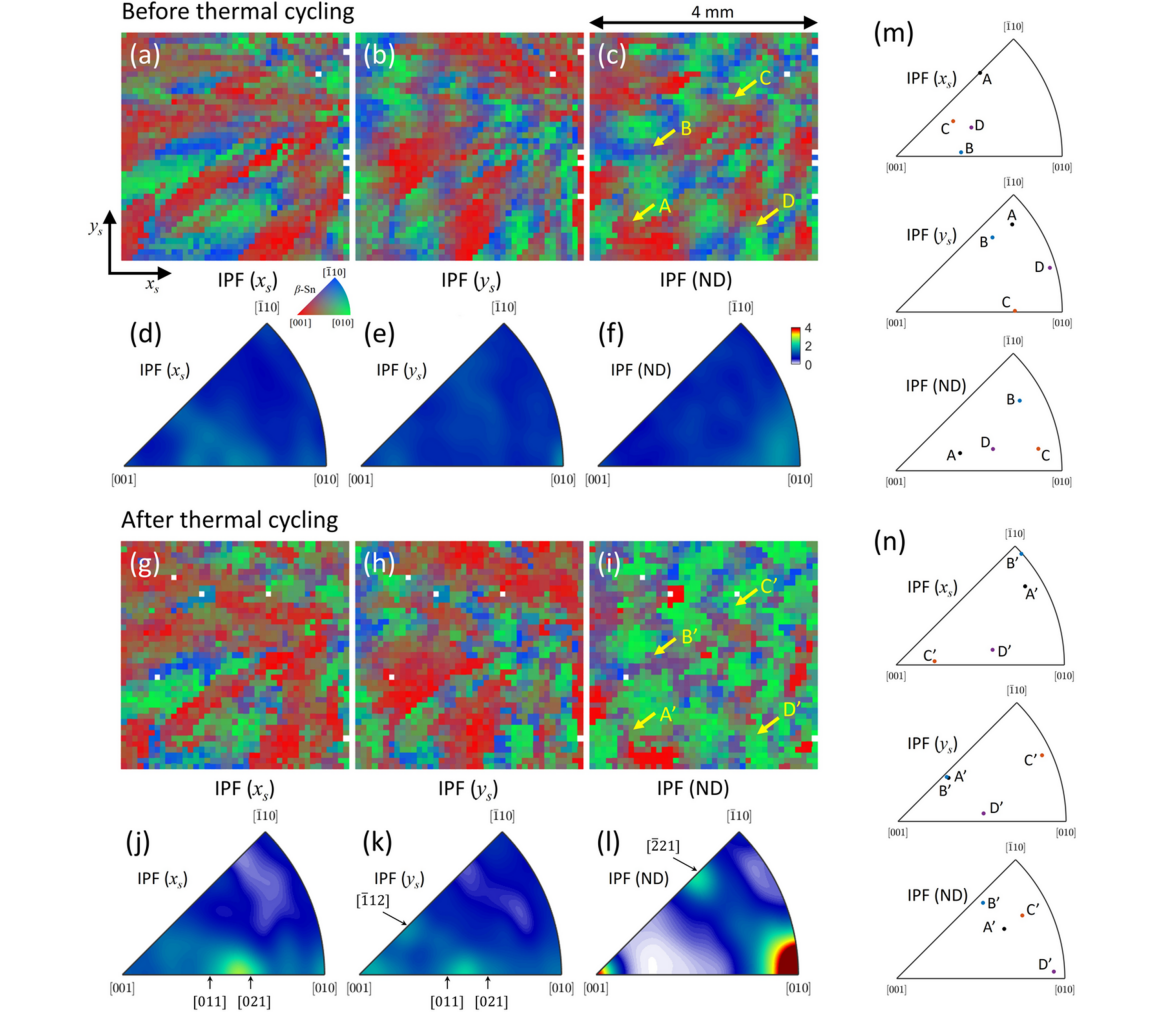
IPF maps and IPF density of β-Sn solder in the second specimen before and after the thermal cycling. IPF (*x*_s_, *y*_s_, ND) maps and their densities before thermal cycling are illustrated in (*a*)–(*f*). IPF (*x*_s_, *y*_s_, ND) maps and their densities after thermal cycling are illustrated in (*g*)–(*l*), respectively. Noticeable orientation changes are observed, especially in the IPF (ND) map. After the thermal cycling test, the *a* axis tended to align parallel to the specimen ND, and smaller fractions of grains exhibited alignment of the *c* axis or the [221] direction parallel to the ND. In the case of IPF (*x*_s_) and IPF(*y*_s_), weak texture components at the [112] direction, and between the [011] and the [021] directions are observed. The orientations of selected individual grains (A, B, C, and D) before and after thermal cycling are shown in (*m*) and (*n*), respectively.

For the second specimen, IPF maps of *x*_s_, *y*_s_, and ND with 100 µm voxel before and after the thermal cycles are illustrated in Fig. 9 ▸. Before thermal cycling, as shown in Figs. 9 ▸(*a*)–9 ▸(*c*), the grain orientations vary gradually across the FoV, similar to the first specimen of pristine. The red color in the IPF (ND) map gradually changed to green, indicating that the *a* axis tended to align with the ND during thermal cycling. To understand the texture change by the thermal cycles more quantitatively, we analyze the IPF density as illustrated in Figs. 9 ▸(*d*)–9 ▸(*f*). We set the scale bar the same to compare the effect of the thermal cycles on the texture. The pristine β-Sn exhibits weak texture in all IPFs, indicating an approximately random orientation distribution. The corresponding IPF scatter plots for the entire area are provided in Fig. S6.

After the thermal cycles (−40°C for 30 min / +175°C 30 min) of 376 times, the IPF maps of the *x*_s_ and *y*_s_ directions show a similar pattern as compared with the pristine specimen with small changes as illustrated in Figs. 9 ▸(*g*) and 9 ▸(*h*). On the other hand, the IPF (ND) map in Fig. 9 ▸(*i*) shows significant changes in the orientation distribution by the thermal cycles. An identical *x*_s_–*y*_s_ plane slice obtained from 3D scan shows results consistent with those obtained from the point-by-point scan, thereby confirming the validity of the point-by-point scan mode, as shown in Fig. S4.

The increase in the green-colored area in the IPF (ND) map in Fig. 9 ▸(*i*) indicates that the crystallographic *a* axis increasingly aligns with the specimen ND during thermal fatigue. The IPF density maps along the specimen *x* and *y* directions [Figs. 9 ▸(*j*) and 9 ▸(*k*)] show enhanced intensities near the [011], [021], and [112] directions. However, because the specimen *x*_s_ and *y*_s_ axes possess rotational degrees of freedom within the sample plane, a detailed interpretation of these components is not straightforward without precise knowledge of the specimen *x*_s_–*y*_s_ coordinate configuration.

In contrast to the IPF (*x*_s_, *y*_s_) densities, the IPF (ND) density shown in Fig. 9 ▸(*l*) exhibits its strongest intensity at [010], consistent with the trend observed in the IPF (ND) maps. In addition, intensity increases near [221] and [001] were observed. The [001] component is confined to a relatively narrow angular range and becomes more pronounced after thermal fatigue. These results indicate that thermal fatigue induces a reorientations of β-Sn grains, resulting in an increased fraction of grains whose *a* axis is parallel to the specimen ND. In addition, the smaller fractions of grains whose *c* axis and [221] directions are parallel to the specimen ND are also increased.

The orientations of individual grains of the second sample before (A, B, C, and D) and after (A′, B′, C′, and D′) thermal cycling are described in Figs. 9 ▸(*m*) and 9 ▸(*n*), respectively. For grains A and B, the specimen ND becomes more closely aligned with the [221] crystallographic direction after thermal cycling. Grain C exhibits a pronounced change in orientation in all directions. For grain D, the *a* axis is nearly parallel to the specimen ND after thermal cycling test, contributing to the enhancement of the [010] corner observed in Fig. 9 ▸(*l*).

**Figure 10:**
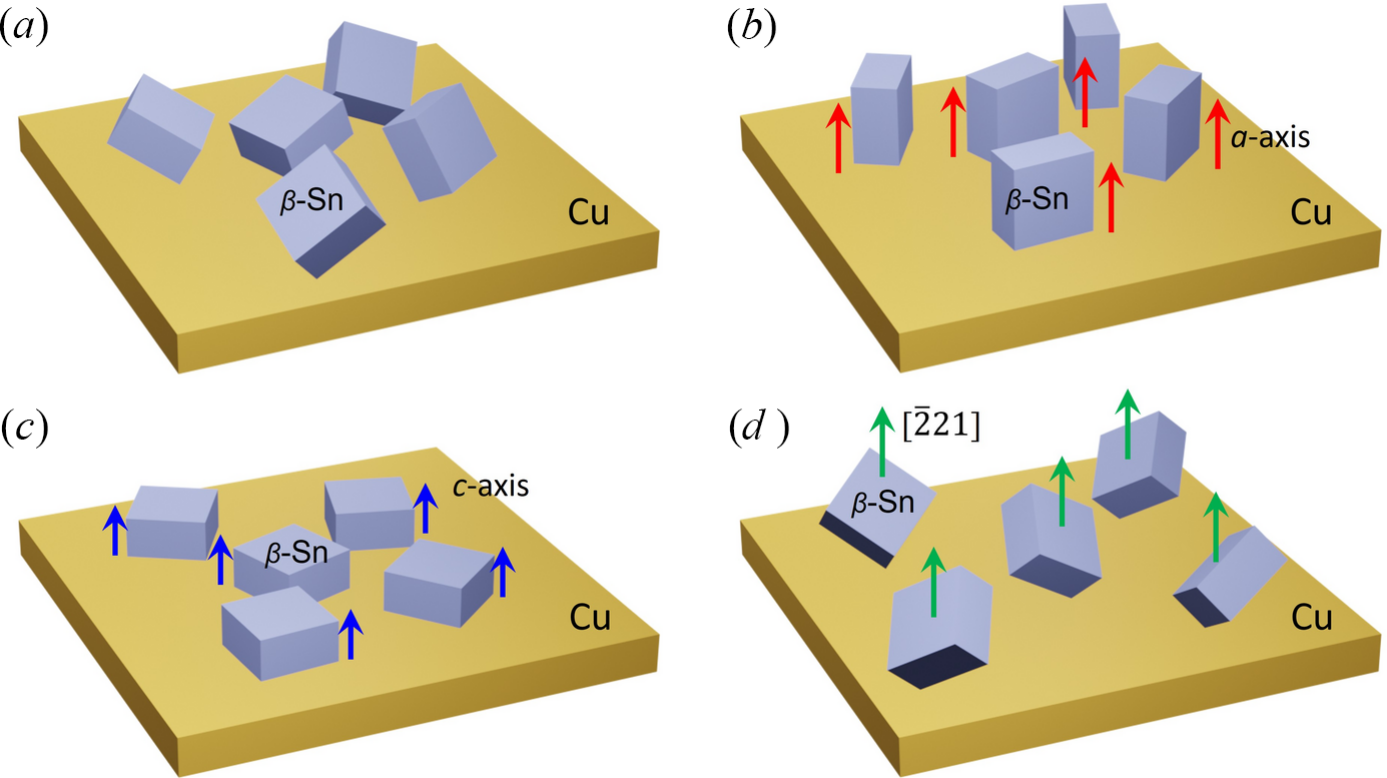
Schematic illustration of the proposed orientation evolution of β-Sn grains during thermal cycling. (*a*) Before thermal cycling, β-Sn grains exhibit an approximately random orientation distribution on the Cu substrate. (*b*) After thermal fatigue, a major fraction of β-Sn grains exhibits orientations in which the *a* axis is parallel to the specimen ND. The red arrows indicate the *a* axis of the β-Sn unit cell. (*c*) For a smaller fraction of β-Sn grains, their *c* axis are parallel to the specimen ND, where the blue arrows indicate the *c* axis of the β-Sn unit cell. (*d*) For another smaller fraction of β-Sn grains, the [221] crystallographic direction is parallel to the specimen ND. The green arrows indicate the [221] directions.

Our observations on the two samples show the reorientation of grains during the thermal cycling tests, as illustrated in Fig. 10 ▸. Before thermal cycling, the β-Sn grains exhibit an approximately random orientation distribution on the Cu substrate, as illustrated schematically in Fig. 10 ▸(*a*). After thermal fatigue, a systematic redistribution of crystallographic orientations is observed, leading to an increased fraction of β-Sn grains whose *a* axis is parallel to the specimen ND, as shown in Fig. 10 ▸(*b*). In addition, smaller fractions of grains exhibit orientations in which the *c* axis or the [221] crystallographic direction is parallel to the specimen ND, as illustrated in Figs. 10 ▸(*c*) and 10 ▸(*d*).

In the first sample, the thermal-cycling temperature was lower than that applied to the second sample, which likely limited the extent of boundary migration. Therefore, the component shown in Fig. 10 ▸(*d*) is dominant under lower-temperature. As a result, the grain reorientation was less pronounced, and the dominant *a* axis alignment with the specimen ND was not fully developed. In contrast, the second sample was subjected to a higher temperature, which likely facilitated grain reorientation through recrystallization accompanied by grain boundary migration and/or coalescence. Consequently, the second sample exhibits pronounced alignment of the *a* axis with the specimen ND. Meanwhile, smaller fractions of grains exhibited orientations in which the *c* axis or the [221] direction was aligned parallel to the specimen ND, as illustrated in Figs. 10 ▸(*b*)–10 ▸(*d*).

## 4. Discussion

The thermal fatigue process progresses through subgrain formation, recrystallization, and crack initiation (Matin *et al.*, 2005 ▸; Bieler *et al.*, 2012 ▸; Zhou *et al.*, 2013 ▸; Han *et al.*, 2017 ▸; Ben Romdhane *et al.*, 2021 ▸; Du *et al.*, 2023 ▸; Wang *et al.*, 2024 ▸). Microstructural evolution is initiated by gradual subgrain reorientation, followed by crack nucleation at high-angle boundaries formed through recrystallization. Due to the anisotropic properties of β-Sn, this process is particularly sensitive to its crystallographic orientation.

Wang *et al.* (2024 ▸) reported that β-Sn shows minimal nucleation and lacks a distinct preferred orientation under normal cooling conditions. Our *i*-S3DXRD results, which show no preferred grain orientation in pristine β-Sn of the specimen, are in good agreement with their findings. One factor affecting solder solidification is the temperature gradient during the bonding process. The variation in thermal dissipation leads to a strong influence on the orientation and configuration of β-Sn grains. Zhao *et al.* (2017 ▸) showed that this gradient plays a key role in the preferred orientation of β-Sn. They found that uniform temperature bonding resulted in randomly distributed β-Sn grains, whereas a temperature gradient caused the *c* axis of β-Sn to align parallel to the Cu/Sn–Ag–Cu/Cu interface in micro-interconnects. Since the *a* and *c* axes are orthogonal in the tetragonal lattice, their observation can equivalently be interpreted as an increased population of β-Sn grains with an *a* axis aligned with the specimen ND. Their EBSD analysis is consistent with our *i*-S3DXRD results, although their focus was on polished surfaces.

A study suggests that alignment of the *c* axis of β-Sn grains with the direction of current flow or a temperature gradient accelerates electromigration (Lu *et al.*, 2008 ▸) and thermomigration (Hsu & Ouyang, 2014 ▸). Experiments (Bieler *et al.*, 2008 ▸; Lee *et al.*, 2010 ▸; Xu *et al.*, 2022 ▸) and modeling (Lövberg *et al.*, 2017 ▸; Ben Romdhane *et al.*, 2020 ▸; Xu *et al.*, 2022 ▸; Xie *et al.*, 2023 ▸) of Sn–Ag–Cu solder joints show that thermal fatigue damage develops more easily in single-grain joints when the *c* axis of β-Sn lies in the plane of the PCB. This is explained by the maximization of the in-plane coefficient of thermal expansion (CTE) mismatch and minimization of out-of-plane CTE, both of which contribute to grain reorientation (Bieler *et al.*, 2008 ▸; Lee *et al.*, 2010 ▸; Lövberg *et al.*, 2017 ▸; Ben Romdhane *et al.*, 2020 ▸; Xu *et al.*, 2022 ▸; Xie *et al.*, 2023 ▸; Xian *et al.*, 2024 ▸).

In the case of BGA joints containing few grains, thermal fatigue damage is increased when the *c* axis of β-Sn is in-plane, maximizing CTE and elasticity mismatches (Bieler *et al.*, 2008 ▸; Xian *et al.*, 2024 ▸). In contrast, the thermal fatigue behavior of die-attach solder joints remains less understood due to the absence of non-destructive, large-area observation techniques (Zhou *et al.*, 2014 ▸; Hosoya *et al.*, 2020 ▸; Sugimoto *et al.*, 2020 ▸; Liu *et al.*, 2024 ▸). Despite observations by EBSD, it remains unclear whether the fatigued structures are thermally induced or pre-existing (Zhou *et al.*, 2013 ▸).

With respect to crystallography, Cu (substrate) has a cubic lattice (*a* = 3.615 Å), whereas β-Sn has a tetragonal lattice. The two structures differ fundamentally, leading to a large lattice mismatch and making epitaxial alignment energetically unfavorable in this system. Therefore, crystallographic matching is unlikely to be the cause of the observed orientation tendency.

It is known that β-Sn has a body-centered tetragonal structure (*c*/*a* = 0.545) and exhibits anisotropic physical properties. For example, the CTEs of β-Sn along the *c* axis are approximately two to three times larger than those along the *a* axis (Matin *et al.*, 2005 ▸; Matin *et al.*, 2007 ▸). The CTE of the *a* and *c* axes of β-Sn at room temperature are 16.5 × 10^−6^ °C^−1^ and 32.4 × 10^−6^ °C^−1^, respectively. At 130 °C, these values increase to 20.2 × 10^−6^ °C^−1^, and 41.2 × 10^−6^ °C^−1^, respectively.

The CTE of the β-Sn *a* axis is comparable with that of Cu (∼16.7 × 10^−6^ °C^−1^; Rubin *et al.*, 1954 ▸), whereas the CTE of β-Sn along the *c* axis is approximately twice that of Cu. From a CTE-mismatch perspective, the *c* axis should be parallel to the ND of the Cu substrate in order to minimize the in-plane CTE mismatch. However, under high-temperature thermal cycling, we observed that the *a* axis of most β-Sn grains tends to align parallel to the ND of the Cu substrate. Equivalently, the *c* axis lies predominantly within the *x*_s_–*y*_s_ plane of the Cu substrate. Under this configuration, the in-plane CTE mismatch is maximized, which may promote the accumulation of in-plane strain during thermal cycling. Therefore, not only the CTE but also other parameters—such as anisotropic elastic constant—may be necessary to describe the grain reorientation associated with recrystallization.

Regarding the anisotropic elastic property, we introduce elastic compliance as an additional parameter to consider in this context. In the case of the elastic moduli (in GPa) of β-Sn, it also shows the clear anisotropic value as follows: *c*_11_ = 73.5, 83.91, 86.0; *c*_12_ = 23.4, 48.70, 35.0; *c*_13_ = 28.0, 28.10, 30.0; *c*_33_ = 87.0, 96.65, 133.0; *c*_44_ = 22.0, 17.54, 49.0; *c*_66_ = 22.65, 7.41, 53.0 (Matin *et al.*, 2005 ▸; Matin *et al.*, 2007 ▸). From these values, we obtain the stiffness matrix (Mouhat & Coudert, 2014 ▸), **C**, as follows
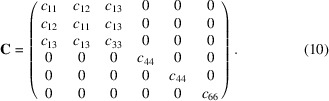
From the inversion of **C**, we obtain the elastic compliance matrix (**S**), which relates applied stress to the resulting elastic strain. The elastic compliance of β-Sn along the *a* axis (*s*_11_) is 0.0163, 0.0185, and 0.0145 GPa^−1^ for the three reported datasets, respectively, whereas the compliance along the *c* axis (*s*_33_) is smaller, with values of 0.0141, 0.0118, and 0.00847 GPa^−1^. This systematic difference (*s*_11_ > *s*_33_) demonstrates that *a* axis of β-Sn is softer than the *c* axis.

Therefore, a plausible explanation is that reorientation during thermal cycling is governed by the combined effects of anisotropic thermal expansion and anisotropic elastic properties of β-Sn under local thermomechanical constraints, which vary with sample position within the joint. Because the elastic compliance is higher along the *a* axis, whereas the thermal expansion coefficient is larger along the *c* axis, different crystal orientations experience distinct strain when constrained. As a result, the *a* axis of the majority of β-Sn grains tends to align parallel to the ND, while a smaller fraction of grains adopts the *c* axis parallel to the ND. In some cases, intermediate orientations, such as near the [221] direction, are also observed to align with the ND. Although this interpretation is not unique, it provides a physically consistent explanation for the coexistence of multiple preferred orientation components observed after thermal cycling.

## 5. Conclusion

In this study, we investigated the orientation evolution of β-Sn grains in die-attach solder joints subjected to thermal cycling tests using *i*-S3DXRD. Orientation maps of β-Sn grains were successfully reconstructed, and good agreement with EBSD measurements confirms the validity of the method. For the first specimen, a 3D scan mode was employed to obtain a high-resolution volumetric orientation map of the β-Sn layer before and after low-temperature thermal cycling. For the second specimen, a point-by-point scan mode was used to acquire a wide-area two-dimensional orientation map before and after high-temperature thermal cycling. We found that the specimen ND tended to align with the [221] direction of β-Sn under the lower-temperature condition. In contrast, under the higher-temperature condition, the *a* axis of β-Sn preferentially aligned parallel to the ND of the Cu substrate, accompanied by an increased fraction of grains whose *c* axis or [221] direction is parallel to the substrate ND. We attribute this grain reorientation to recrystallization accompanied by grain boundary migration and grain coalescence during thermal cycling.

## Supplementary Material

Supplementary information. DOI: 10.1107/S1600577526001475/tol5020sup1.pdf

VideoS1. DOI: 10.1107/S1600577526001475/tol5020sup2.avi

VideoS2. DOI: 10.1107/S1600577526001475/tol5020sup3.avi

## Data Availability

The data are available from the corresponding author upon reasonable request.

## References

[bb1] Adams, B. L. (1997). *Ultramicroscopy* **67**, 11–17.

[bb2] Alabort, E., Kontis, P., Barba, D., Dragnevski, K. & Reed, R. C. (2016). *Acta Mater.* **105**, 449–463.

[bb3] Barabash, R. I., Ice, G. E., Liu, W. & Barabash, O. M. (2009). *Micron* **40**, 28–36. 10.1016/j.micron.2008.03.010 18472271

[bb4] Ben Romdhane, E., Guédon-Gracia, A., Pin, S., Roumanille, P. & Frémont, H. (2020). *Microelectron. Reliab.* **114**, 113812.

[bb5] Ben Romdhane, E., Roumanille, P., Guédon-Gracia, A., Pin, S., Nguyen, P. & Frémont, H. (2021). *Microelectron. Reliab.* **126**, 114288.

[bb6] Bernier, J. V., Barton, N. R., Lienert, U. & Miller, M. P. (2011). *J. Strain Anal. Eng. Des.* **46**, 527–547.

[bb7] Bieler, T. R., Jiang, H., Lehman, L. P., Kirkpatrick, T., Cotts, E. J. & Nandagopal, B. (2008). *IEEE Trans. C. Packag. Technol.* **31**, 370–381.

[bb8] Bieler, T. R., Zhou, B., Blair, L., Zamiri, A., Darbandi, P., Pourboghrat, F., Lee, T. & Liu, K. (2012). *J. Elec Mater.* **41**, 283–301.

[bb9] Borbely, A., Renversade, L., Kenesei, P. & Wright, J. (2014). *J. Appl. Cryst.* **47**, 1042–1053.

[bb10] Boyce, B. L., Clark, B. G., Lu, P., Carroll, J. D. & Weinberger, C. R. (2013). *Metall. Mater. Trans. A* **44**, 4567–4580.

[bb11] Carlsen, M., Appel, C., Hearn, W., Olsson, M., Menzel, A. & Liebi, M. (2024). *J. Appl. Cryst.* **57**, 986–1000. 10.1107/S1600576724004588 PMC11299598 39108827

[bb12] Cheng, S., Huang, C. & Pecht, M. (2017). *Microelectron. Reliab.* **75**, 77–95.

[bb13] Cnudde, V. & Boone, M. N. (2013). *Earth Sci. Rev.* **123**, 1–17.

[bb14] Du, Y., Wang, Y., Ji, X., Tan, S., Han, J. & Guo, F. (2023). *Mater. Charact.* **200**, 112890.

[bb15] Frewein, M. P. K., Mason, J., Maier, B., Cölfen, H., Medjahed, A., Burghammer, M., Allain, M. & Grünewald, T. A. (2024). *IUCrJ* **11**, 809–820. 10.1107/S2052252524006547 PMC11364025 39046078

[bb16] Gholinia, A., Donoghue, J., Garner, A., Curd, M., Lawson, M. J., Winiarski, B., Geurts, R., Withers, P. J. & Burnett, T. L. (2024). *Ultramicroscopy* **257**, 113903. 10.1016/j.ultramic.2023.113903 38101083

[bb17] Gondrom, S., Zhou, J., Maisl, M., Reiter, H., Kröning, M. & Arnold, W. (1999). *Nucl. Eng. Des.* **190**, 141–147.

[bb18] Han, J., Guo, F. & Liu, J. P. (2017). *J. Alloys Compd.* **698**, 706–713.

[bb19] Hayashi, Y., Hirose, Y. & Seno, Y. (2015). *J. Appl. Cryst.* **48**, 1094–1101.

[bb20] Hayashi, Y., Setoyama, Y., Hirose, Y., Yoshida, T. & Kimura, T. (2019). *Science* **366**, 1492–1496. 10.1126/science.aax9167 31857480

[bb22] Hektor, J., Hall, S. A., Henningsson, N. A., Engqvist, J., Ristinmaa, M., Lenrick, F. & Wright, J. P. (2019). *Materials* **12**, 446. 10.3390/ma12030446 PMC6384662 30709058

[bb23] Henningsson, A., Kutsal, M., Wright, J. P., Ludwig, W., Sørensen, H. O., Hall, S. A., Winther, G. & Poulsen, H. F. (2024). *Sci. Rep.* **14**, 20213. 10.1038/s41598-024-71006-0 PMC11364660 39215107

[bb24] Henningsson, N. A., Hall, S. A., Wright, J. P. & Hektor, J. (2020). *J. Appl. Cryst.* **53**, 314–325. 10.1107/S1600576720001016 PMC7133059 32280319

[bb25] Hoshino, M., Uesugi, K., Takeuchi, A., Suzuki, Y., Yagi, N., McNulty, I., Eyberger, C. & Lai, B. (2011). *AIP Conf. Proc.* **1365**, 250–253.

[bb26] Hosoya, K., Kariya, Y., Sugimoto, H. & Takahashi, K. (2020). *J. Electron. Mater.* **49**, 6175–6186.

[bb27] Hsu, W.-N. & Ouyang, F.-Y. (2014). *Acta Mater.* **81**, 141–150.

[bb28] Humphreys, F. J. (2001). *J. Mater. Sci.* **36**, 3833–3854.

[bb29] Ice, G. E., Budai, J. D. & Pang, J. W. L. (2011). *Science* **334**, 1234–1239. 10.1126/science.1202366 22144618

[bb30] Johnson, G., King, A., Honnicke, M. G., Marrow, J. & Ludwig, W. (2008). *J. Appl. Cryst.* **41**, 310–318.

[bb31] Juul, N. Y., Oddershede, J., Beaudoin, A., Chatterjee, K., Koker, M. K. A., Dale, D., Shade, P. & Winther, G. (2017). *Acta Mater.* **141**, 388–404.

[bb32] Juul, N. Y., Oddershede, J. & Winther, G. (2020). *JOM* **72**, 83–90.

[bb33] Kalender, W. A. (2006). *Phys. Med. Biol.* **51**, R29–R43. 10.1088/0031-9155/51/13/R03 16790909

[bb34] Kim, J., Hayashi, Y., Ha, S. S. & Yabashi, M. (2026). *Comput. Phys. Commun.* **320**, 109988.

[bb36] Kim, J., Hayashi, Y. & Yabashi, M. (2023*a*). *J. Synchrotron Rad.* **30**, 1108–1113. 10.1107/S1600577523008597 PMC10624026 37850563

[bb35] Kim, J., Hayashi, Y. & Yabashi, M. (2023*b*). *J. Appl. Cryst.* **56**, 1416–1425.

[bb37] Larson, B. C. & Levine, L. E. (2013). *J. Appl. Cryst.* **46**, 153–164.

[bb38] Larson, B. C., Yang, W., Ice, G. E., Budai, J. D. & Tischler, J. Z. (2002). *Nature* **415**, 887–890. 10.1038/415887a 11859363

[bb39] Lasalmonie, A. & Strudel, J. L. (1986). *J. Mater. Sci.* **21**, 1837–1852.

[bb40] Lee, T., Zhou, B., Blair, L. K. L., Liu, K. & Bieler, T. R. (2010). *J. Electon. Mater.* **39**, 2588–2597.

[bb41] Levine, L. E., Larson, B. C., Yang, W., Kassner, M. E., Tischler, J. Z., Delos-Reyes, M. A., Fields, R. J. & Liu, W. (2006). *Nat. Mater.* **5**, 619–622. 10.1038/nmat1698 16845413

[bb42] Li, S. F., Lind, J., Hefferan, C. M., Pokharel, R., Lienert, U., Rollett, A. D. & Suter, R. M. (2012). *J. Appl. Cryst.* **45**, 1098–1108.

[bb43] Lienert, U., Li, S. F., Hefferan, C. M., Lind, J., Suter, R. M., Bernier, J. V., Barton, N. R., Brandes, M. C., Mills, M. J., Miller, M. P., Jakobsen, B. & Pantleon, W. (2011). *JOM* **63**, 70–77.

[bb21] Lifeng He,, Yuyan Chao, & Suzuki, K. (2011). *IEEE Trans. Image Process.* **20**, 2122–2134. 10.1109/TIP.2011.2114352 PMC4283815 21324785

[bb44] Liu, S., Vuorinen, V., Liu, X., Fredrikson, O., Brand, S., Tiwary, N., Lutz, J. & Paulasto-Kröckel, M. (2024). *IEEE Trans. Power Electron.* **39**, 16695–16707.

[bb45] Lövberg, A., Tegehall, P., Wetter, G., Brinkfeldt, K. & Andersson, D. (2017). *Proceedings of the 18th International Conference on Thermal, Mechanical and Multi-Physics Simulation and Experiments in Microelectronics and Microsystems (EuroSimE2017)*.

[bb46] Lu, M., Shih, D., Lauro, P., Goldsmith, C. & Henderson, D. W. (2008). *Appl. Phys. Lett.* **92**, 211909.

[bb47] Ludwig, W., King, A., Reischig, P., Herbig, M., Lauridsen, E. M., Schmidt, S., Proudhon, H., Forest, S., Cloetens, P., Roscoat, S. R., Buffière, J. Y., Marrow, T. J. & Poulsen, H. F. (2009). *Mater. Sci. Eng. A* **524**, 69–76.

[bb48] Ludwig, W., Schmidt, S., Lauridsen, E. M. & Poulsen, H. F. (2008). *J. Appl. Cryst.* **41**, 302–309.

[bb49] MacSleyne, J., Uchic, M. D., Simmons, J. P. & De Graef, M. (2009). *Acta Mater.* **57**, 6251–6267.

[bb50] Manikam, V. R. & Cheong, K. Y. (2011). *IEEE Trans. Compon. Packag. Manuf. Technol.* **1**, 457–478.

[bb51] Margulies, L., Winther, G. & Poulsen, H. F. (2001). *Science* **291**, 2392–2394. 10.1126/science.1057956 11264531

[bb52] Matin, M. A., Coenen, E. W. C., Vellinga, W. P. & Geers, M. G. D. (2005). *Scr. Mater.* **53**, 927–932.

[bb53] Matin, M. A., Vellinga, W. P. & Geers, M. G. D. (2007). *Mater. Sci. Eng. A* **445–446**, 73–85.

[bb54] Mayo, M. J. & Nix, W. D. (1989). *Acta Metall.* **37**, 1121–1134.

[bb55] Moore, T. D., Vanderstraeten, D. & Forssell, P. M. (2002). *IEEE Trans. C. Packag. Technol.* **25**, 224–229.

[bb56] Mouhat, F. & Coudert, F.-X. (2014). *Phys. Rev. B* **90**, 224104.

[bb57] Oddershede, J., Sun, J., Gueninchault, N., Bachmann, F., Bale, H., Holzner, C. & Lauridsen, E. (2019). *Integr. Mater. Manuf. Innov.* **8**, 217–225.

[bb58] Pineau, A., McDowell, D. L., Busso, E. P. & Antolovich, S. D. (2016). *Acta Mater.* **107**, 484–507.

[bb59] Pokharel, R., Lind, J., Kanjarla, A. K., Lebensohn, R. A., Li, S. F., Kenesei, P., Suter, R. M. & Rollett, A. D. (2014). *Annu. Rev. Condens. Matter Phys.* **5**, 317–346.

[bb60] Poulsen, H. F. (2012). *J. Appl. Cryst.* **45**, 1084–1097.

[bb61] Poulsen, H. F. & Fu, X. (2003). *J. Appl. Cryst.* **36**, 1062–1068.

[bb62] Poulsen, H. F., Margulies, L., Schmidt, S. & Winther, G. (2003). *Acta Mater.* **51**, 3821–3830.

[bb63] Poulsen, H. F., Nielsen, S. F., Lauridsen, E. M., Schmidt, S., Suter, R. M., Lienert, U., Margulies, L., Lorentzen, T. & Juul Jensen, D. (2001). *J. Appl. Cryst.* **34**, 751–756.

[bb64] Regalado, I. L., Williams, J. J., Joshi, S., Dede, E. M., Liu, Y. & Chawla, N. (2019). *Adv. Eng. Mater.* **21**, 1801029.

[bb65] Reischig, P., King, A., Nervo, L., Viganó, N., Guilhem, Y., Palenstijn, W. J., Batenburg, K. J., Preuss, M. & Ludwig, W. (2013). *J. Appl. Cryst.* **46**, 297–311.

[bb66] Rowenhorst, D. J., Lewis, A. C. & Spanos, G. (2010). *Acta Mater.* **58**, 5511–5519.

[bb67] Rubin, T., Altman, H. W. & Johnston, H. L. (1954). *J. Am. Chem. Soc.* **76**, 5289–5293.

[bb68] Schmidt, S. (2014). *J. Appl. Cryst.* **47**, 276–284.

[bb69] Seo, S., Kang, S. K., Shih, D. & Lee, H. M. (2009). *J. Electron. Mater.* **38**, 257–265.

[bb70] Sharma, H., Huizenga, R. M. & Offerman, S. E. (2012). *J. Appl. Cryst.* **45**, 693–704.

[bb71] Sugimoto, H., Kariya, Y., Abe, Y., Hanada, R., Yokoyama, Y. & Soda, S. (2020). *J. Smart Process.* **9**, 224.

[bb72] Sun, J., Dake, J. M. & Oddershede, J. (2024). *Tomogr. Mater. Struct.* **4**, 100025.

[bb73] Tatsumi, H., Moon, S., Takahashi, M., Kozawa, T., Tsushima, E. & Nishikawa, H. (2024). *Mater. Des.* **238**, 112637.

[bb74] Wang, J., Wang, X., Lv, Z., Zhang, L., Wang, J., Zhang, W., Chen, H. & Li, M. (2024). *Mater. Today Commun.* **38**, 107776.

[bb75] Winther, G., Wright, J. P., Schmidt, S. & Oddershede, J. (2017). *Int. J. Plast.* **88**, 108–125.

[bb76] Wright, S. I., Nowell, M. M. & Field, D. P. (2011). *Microsc. Microanal.* **17**, 316–329. 10.1017/S1431927611000055 21418731

[bb77] Xian, J. W., Xu, Y. L., Stoyanov, S., Coyle, R. J., Dunne, F. P. E. & Gourlay, C. M. (2024). *Nat. Commun.* **15**, 4258. 10.1038/s41467-024-48532-6 PMC11106336 38769155

[bb78] Xie, M., Chen, G., Yuan, X., Zhang, L. & Lin, Q. (2023). *J. Mater. Res. Technol.* **27**, 7195–7212.

[bb79] Xu, Y., Xian, J., Stoyanov, S., Bailey, C., Coyle, R. J., Gourlay, C. M. & Dunne, F. P. E. (2022). *Int. J. Plast.* **155**, 103308.

[bb80] Yazdan Mehr, M., Bahrami, A., Fischer, H., Gielen, S., Corbeij, R., van Driel, W. D. & Zhang, G. Q. (2015). *Proceedings of the 16th International Conference on Thermal, Mechanical and Multi-Physics Simulation and Experiments in Microelectronics and Microsystems (EuroSimE2015)*, April 2015, Budapest, Hungary.

[bb81] Yi, Q., Li, G., Zhang, J., Luo, S.-N., Fan, D., Gao, Z., Wang, Y., Gao, G., Jiang, S. & Jiang, X. (2015). *J. Synchrotron Rad.* **22**, 1062–1071. 10.1107/S1600577515006165 26134812

[bb82] Yu, T., Hong, C., Zhang, Y., Lindkvist, A., Liu, W., Tischler, J. & Jensen, D. J. (2023). *Mater. Charact.* **202**, 112997.

[bb83] Zhao, N., Zhong, Y., Dong, W., Huang, M. L., Ma, H. T. & Wong, C. P. (2017). *Appl. Phys. Lett.* **110**, 093504.

[bb84] Zhou, B., Bieler, T. R., Lee, T. & Liu, W. (2013). *J. Electron. Mater.* **42**, 319–331.

[bb85] Zhou, B., Muralidharan, G., Kurumadalli, K., Parish, C. M., Leslie, S. & Bieler, T. R. (2014). *J. Electron Mater.* **43**, 57–68.

