# Supplementary material for: Non-destructive orientation tracking of individual β-Sn grains in die-attach solder joints
Source: J Synchrotron Radiat. 2026 Mar 20;33(Pt 3):617–31. doi: 10.1107/S1600577526001475 (PMC13148611; doi:10.1107/S1600577526001475)
Supplement: Supplementary file 1 [file s-33-00617-sup1.pdf]

JOURNAL OF SYNCHROTRON RADIATION

Volume 33 (2026)

Supporting information for article:

Non-destructive orientation tracking of individual β-Sn grains in die-attach solder joints

Jaemyung Kim, Yujiro Hayashi, Hiroaki Tatsumi, Hiroshi Nishikawa and Makina Yabashi

Supplementary information for

Non-destructive orientation tracking of individual *β*-Sn grains in die-attach solder joints

Jaemyung Kim[1*], Yujiro Hayashi[1,2], Hiroaki Tatsumi[3], Hiroshi Nishikawa[3], and Makina Yabashi[1,2]

1. RIKEN SPring-8 Center, 1-1-1 Kouto, Sayo-cho, Sayo-gun, Hyogo, 679-5148, Japan
2. Japan Synchrotron Radiation Research Institute, Sayo-cho, Sayo-gun, Hyogo, 679-5148, Japan
3. Joining and Welding Research Institute, Osaka University, 11-1 Mihogaoka, Ibaraki, Osaka, 567-0047, Japan

Quantifying angular loss due to X-ray absorption in vertical rotation geometry 3

Supplementary Figures

Fig. S1. Representative raw diffraction images of the specimen 5

Fig. S2. Distribution of voxel–voxel neighbor misorientations before and after the thermal cycling test 6

Fig. S3. Calibrated 2θ–η map obtained using diffraction patterns from a *β*-Sn 7

Fig. S4. IPF maps of Sn solder after thermal cycle fatigue reconstructed from the 3D scan mode and point-by-point scan mode 8

Fig. S5. IPF of the first sample with a scatter plot. 9

Fig. S6. IPF of the second sample with a scatter plot 10

Fig. S7. IPF maps of the first specimen at z = -40μm before thermal cycling tests 11

Fig. S8. IPF maps of the first specimen at z = -20μm before thermal cycling tests 12

Fig. S9. IPF maps of the first specimen at z = 0μm before thermal cycling tests 13

Fig. S10. IPF maps of the first specimen at z = +20μm before thermal cycling tests 14

Fig. S11. IPF maps of the first specimen at z = +40μm before thermal cycling tests 15

Fig. S12. IPF maps of the first specimen at z = -40μm after thermal cycling tests 16

Fig. S13. IPF maps of the first specimen at z = -20μm after thermal cycling tests 17

Fig. S14. IPF maps of the first specimen at z = 0μm after thermal cycling tests 18

Fig. S15. IPF maps of the first specimen at z = +20μm after thermal cycling tests 19

Fig. S16. IPF maps of the first specimen at z = +40μm after thermal cycling tests 20

Supplementary Videos

Video. S1. 3D IPF (x_s, y_s, ND) maps of the first specimen before thermal cycling 21

Video. S2. 3D IPF (x_s, y_s, ND) maps of the first specimen after thermal cycling 22

Quantifying angular loss due to X-ray absorption in vertical rotation geometry

We evaluated the effect of the missing angular range by comparing the detectable diffraction intensity in the inclined and non-inclined geometries. In our experiment, the intensity threshold for successful reconstruction was approximately 300 counts for a 150 ms exposure with a 45° inclined rotation axis. This threshold is slightly above the noise level of the flat-panel detector (Varex 4343CT).

For comparison with a non-inclined (vertical-rotation) geometry, the effective X-ray path length through the solder layer increases by a factor of 1/cos(45∘). The mass attenuation coefficient of Sn at 37 keV is (μ/ρ) = 23.84cm^2/g (NIST database). Using the density of the tetragonal phase β-Sn, ρ=7.31 g/cm^3, the linear attenuation coefficient is

$$\mu = (\mu/\rho) \times \rho = 23.84 \times 7.31 = 174.2\ \mathrm{cm}^{-1}.$$

For a solder-layer thickness of t = 110 μm = 0.011 cm, the transmission at normal incidence (T) is

$$T = \exp(-\mu t) = \exp(-174.2 \times 0.011) = \exp(-1.916) = 0.147$$

which means that only about 15% of the 37 keV beam reaches the detector when the beam is perpendicular to the Sn layer.

In i-S3DXRD (inclined laminography), the effective material thickness is $t/\cos\theta$, where θ is the inclination angle. In our experiment, the reconstruction threshold of 300 counts satisfies

$$300 = I_0 \exp(-\mu t\ /\ \cos 45°)$$

from which the effective unattenuated intensity (I_0) is obtained as 4507 counts.

To determine the limiting inclination angle at which the detected signal falls to the noise level of the detector (≈100 counts), we solve

$$100 = 4507 \exp(-1.916 / \cos\theta).$$

This yields θ=60°. Thus, the sample becomes effectively opaque for θ>60°. Because rotation passes through both $+\theta$ and $-\theta$, the unavailable angular range is 120°.

This result demonstrates that conventional vertical-rotation geometry is not suitable for planar specimens, as a substantial portion of the rotation range becomes unusable due to strong X-ray absorption in the solder layer. This also implies that approximately one-third of the diffraction information (120° / 360°) may be missing, which inevitably leads to reconstruction errors.

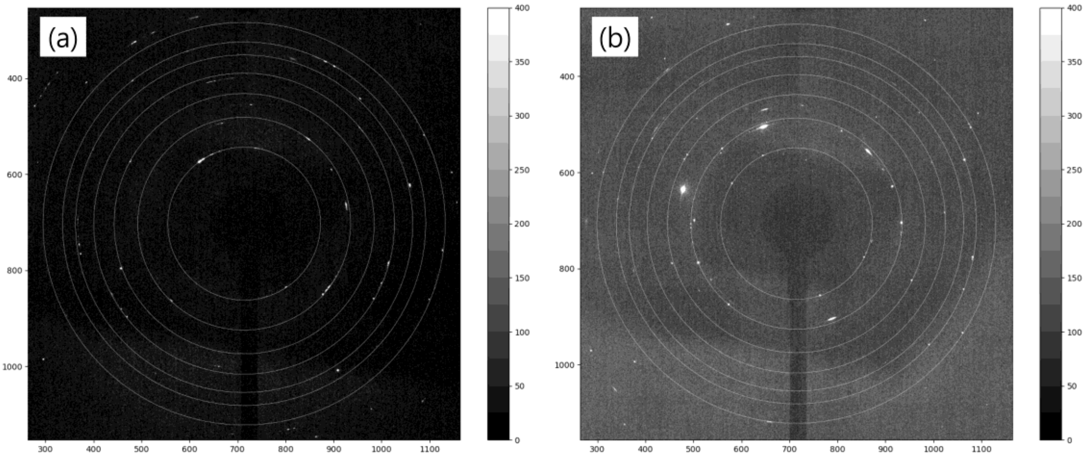

Fig. S1. Representative raw diffraction images of the specimen. (a) before and (b) after the thermal cycling test, shown without any artificial filtering. The background level is higher in (b) due to measurement-induced fluctuations. The seven Debye–Scherrer rings correspond to the tetragonal *β*-Sn reflections (101), (211), (301), (321), (411), (312), and (431), while additional peaks originate from other layers in the multilayer specimen under 37 keV illumination. In some *β*-Sn rings, the diffraction intensity is azimuthally elongated. This azimuthal broadening indicates the presence of smooth orientation gradients within certain grains, whereas other grains show more localized, spot-like intensities. These features are consistent with the intragranular orientation variations observed in the reconstructed maps.

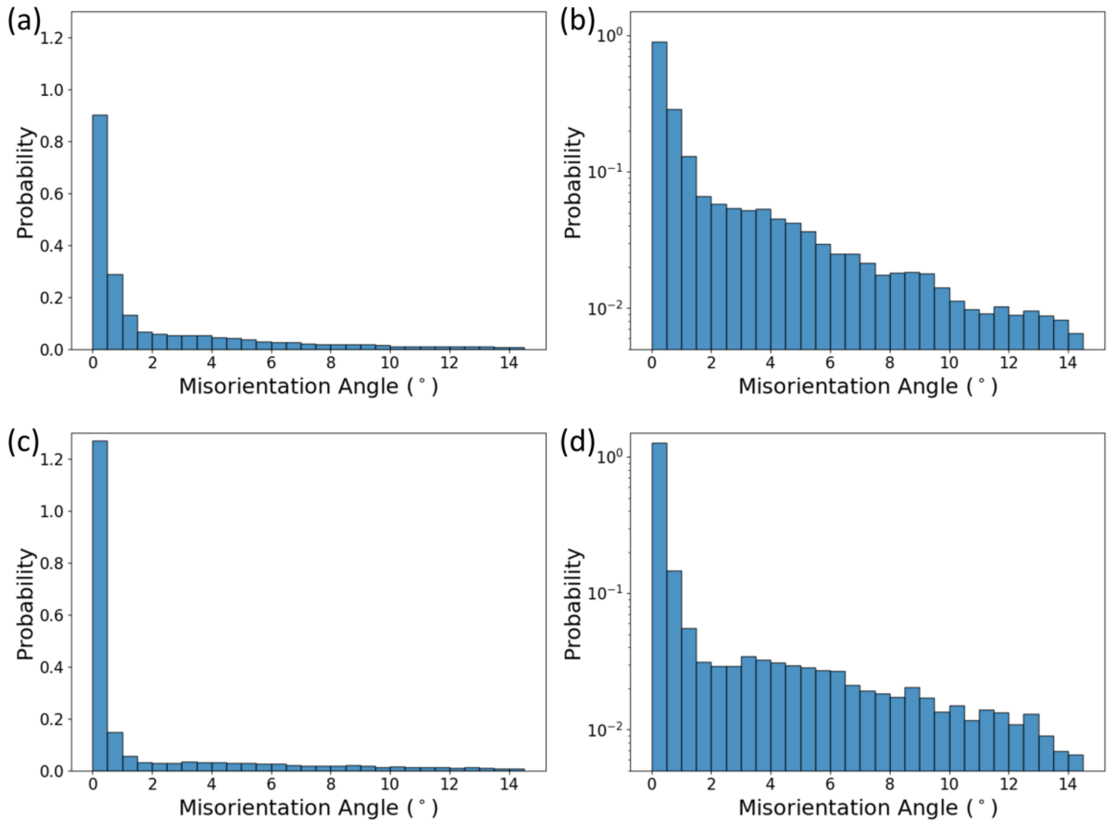

Fig. S2. Distribution of voxel–voxel neighbor misorientations before and after the thermal cycling test. (a) and (b) show the probability density on linear and logarithmic scales, respectively, before thermal cycling. (c) and (d) present the corresponding distributions after thermal cycling. After thermal cycling, the increased fraction of low-angle misorientations indicates a reduction in intra-granular misorientation, consistent with grain growth or recovery processes. This indicates that the 1° threshold effectively separates physically meaningful grain boundaries from intra-granular orientation variations, and does not introduce over-segmentation.

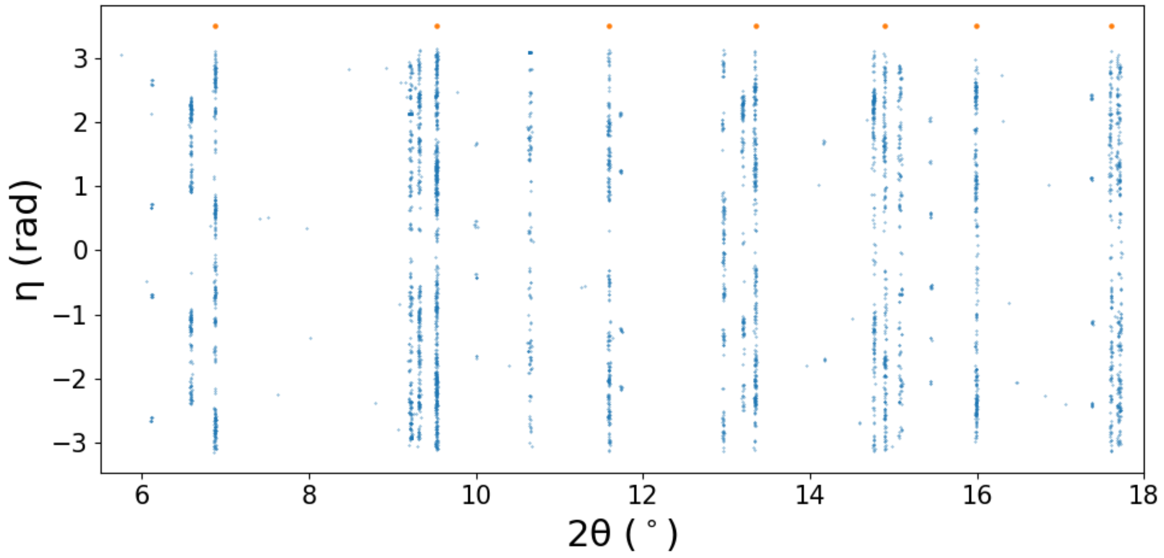

Fig. S3. Calibrated 2θ–η map obtained using diffraction patterns from a β-Sn. The straightening of the diffraction rings confirms that the detector tilt was successfully corrected. The calibrated detector parameters (sample–detector distance, detector center, and detector tilt) were used for all subsequent reconstructions. The red dots indicate the β-Sn diffraction peak positions corresponding to the (101), (211), (301), (321), (411), (312), and (431) reflections.

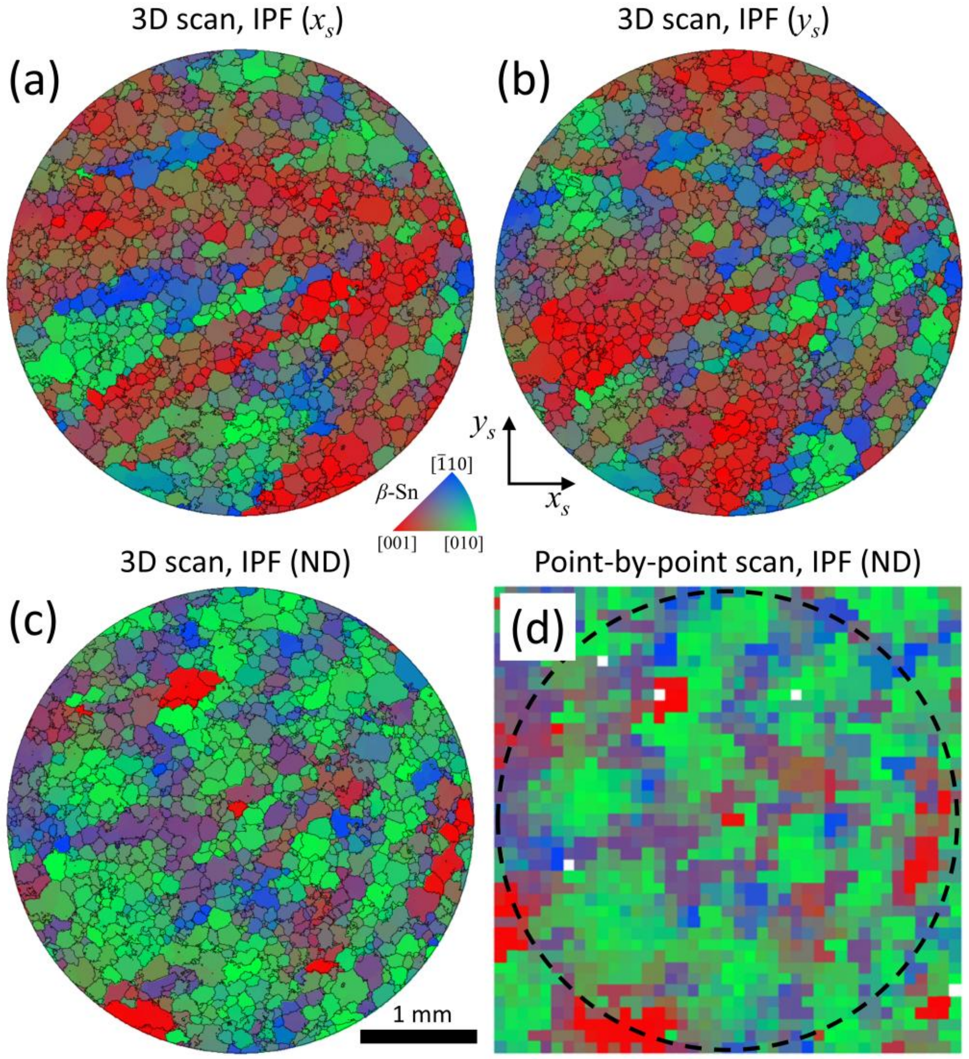

Fig. S4. IPF maps of Sn solder after thermal cycle fatigue reconstructed from the 3D scan mode and point-by-point scan mode. (a-c) The reconstructed orientation map from the 3D scan mode shows clear grain boundaries due to the small voxel size of 10 μm, which is achieved by the super-resolution technique considering the X-ray beam trajectories. (d) IPF (ND) map after the fatigue shows a much larger voxel size compared to that from 3D scan mode. The dashed circle corresponds to the circumference of the IPF (ND) map from 3D scan mode. The color of each voxel is highly matched with that measured by point-by-point scan mode.

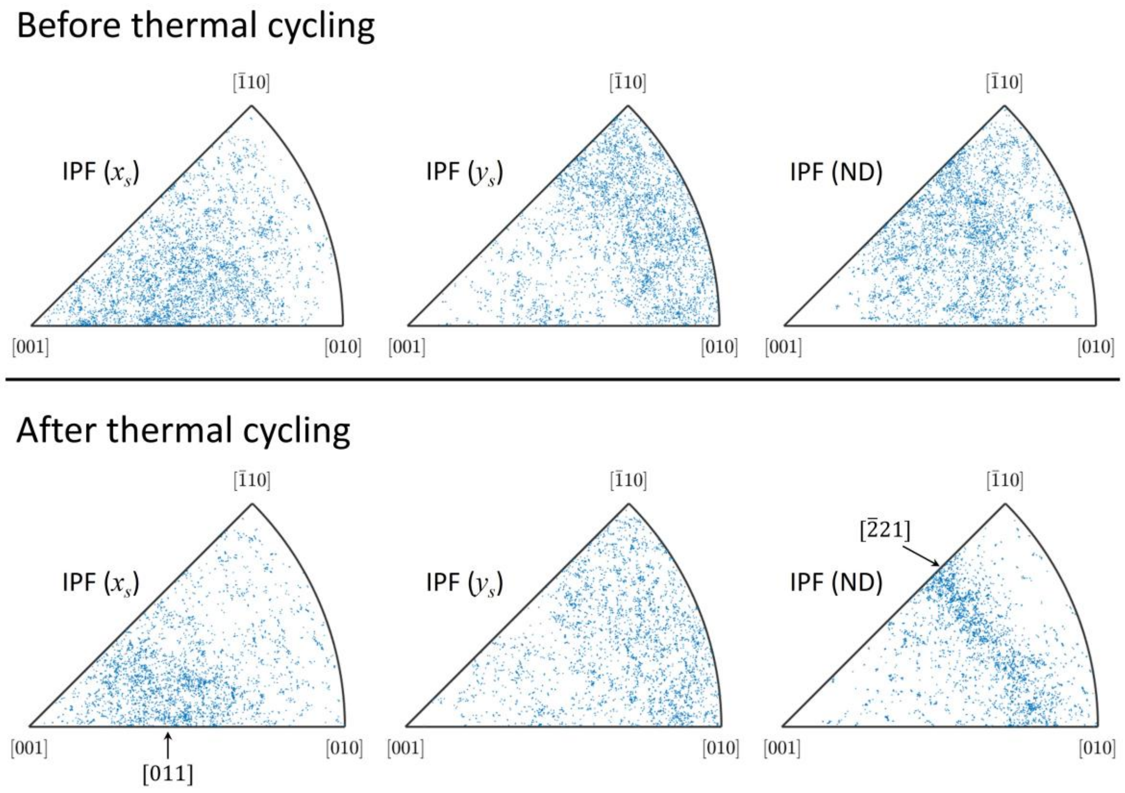

Fig. S5. IPF of the first sample with a scatter plot. Each dot is from the IPF map shown in Fig. 2 of the main text. The IPFs before and after thermal cycling are consistent with the IPF density map.

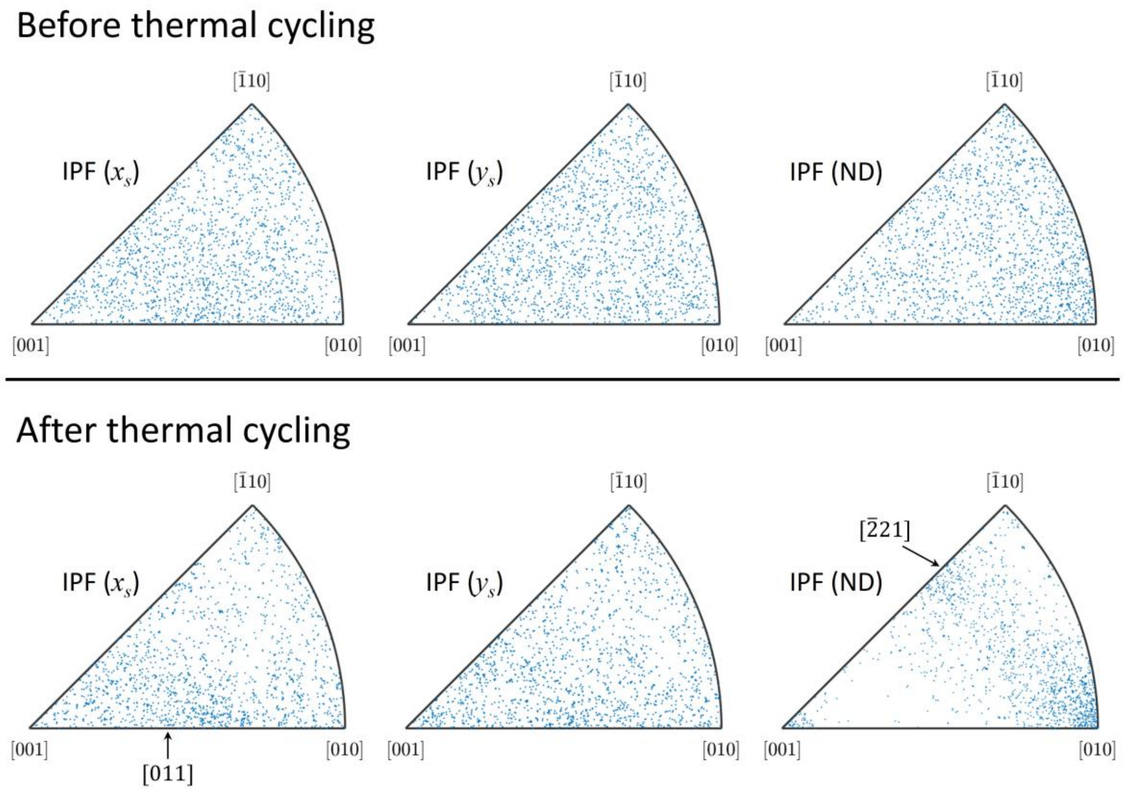

Fig. S6. IPF of the second sample with a scatter plot. Each dot is from the IPF map shown in Fig. 4 of the main text. The IPFs before and after thermal cycling are consistent with the IPF density map.

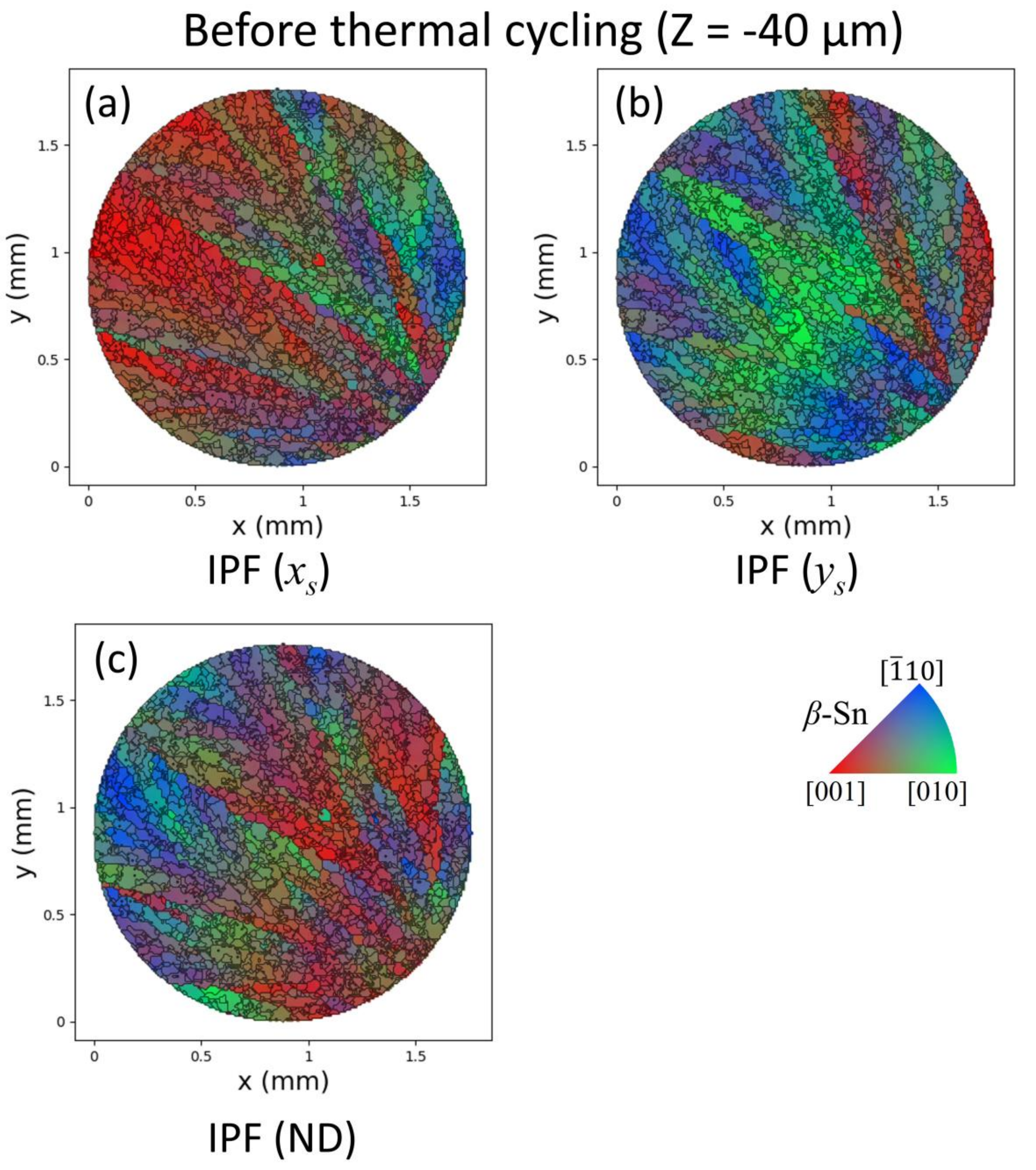

Fig. S7. IPF maps of the first specimen at z = - 40µm before thermal cycling tests. (a-c) IPF (x_s), IPF (y_s), and IPF (ND) maps are illustrated with the grain boundaries of the 1° misorientation threshold.

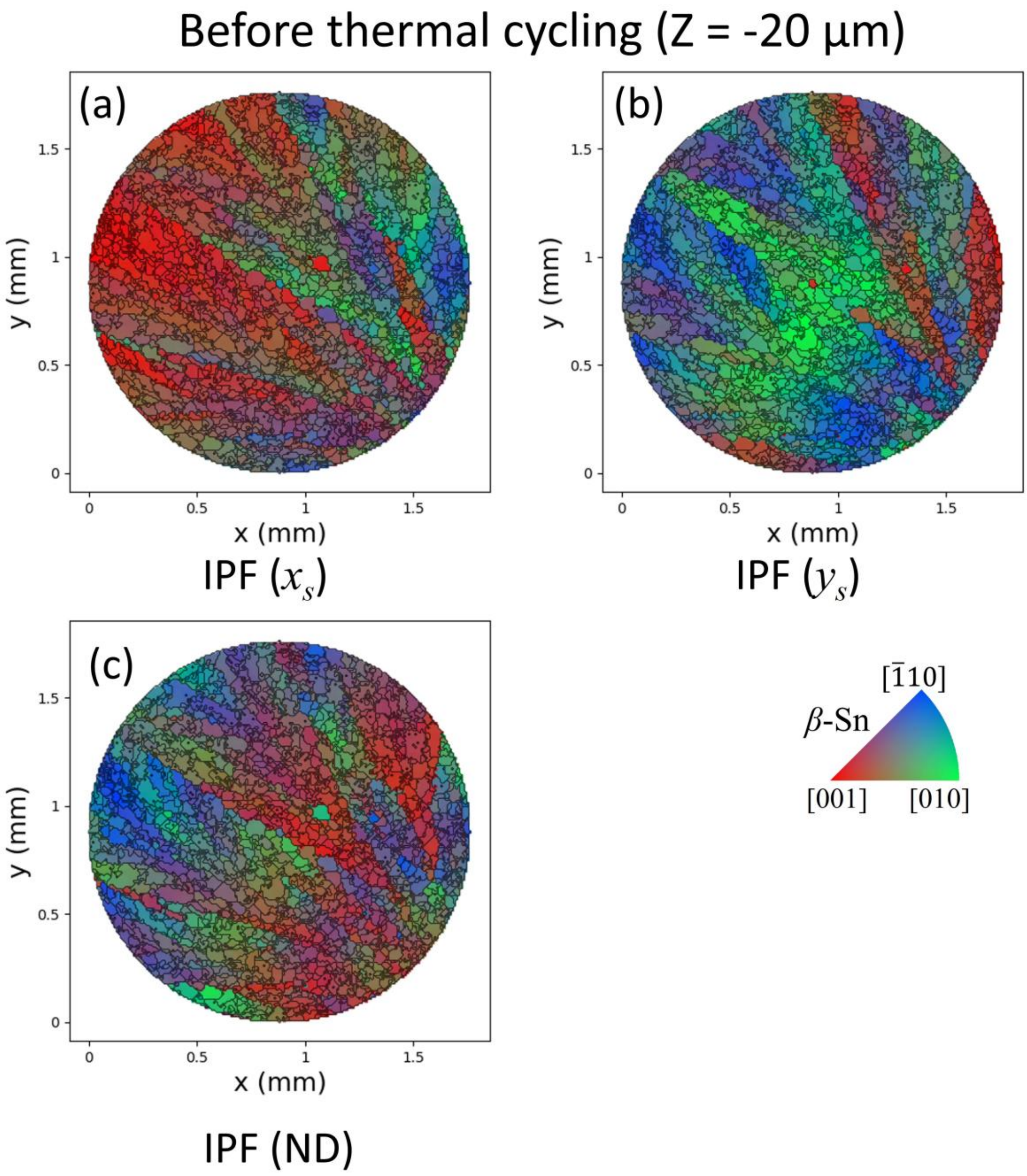

Fig. S8. IPF maps of the first specimen at z = -20μm before thermal cycling tests. (a-c) IPF (x_s), IPF (y_s), and IPF (ND) maps are illustrated with the grain boundaries of the 1° misorientation threshold.

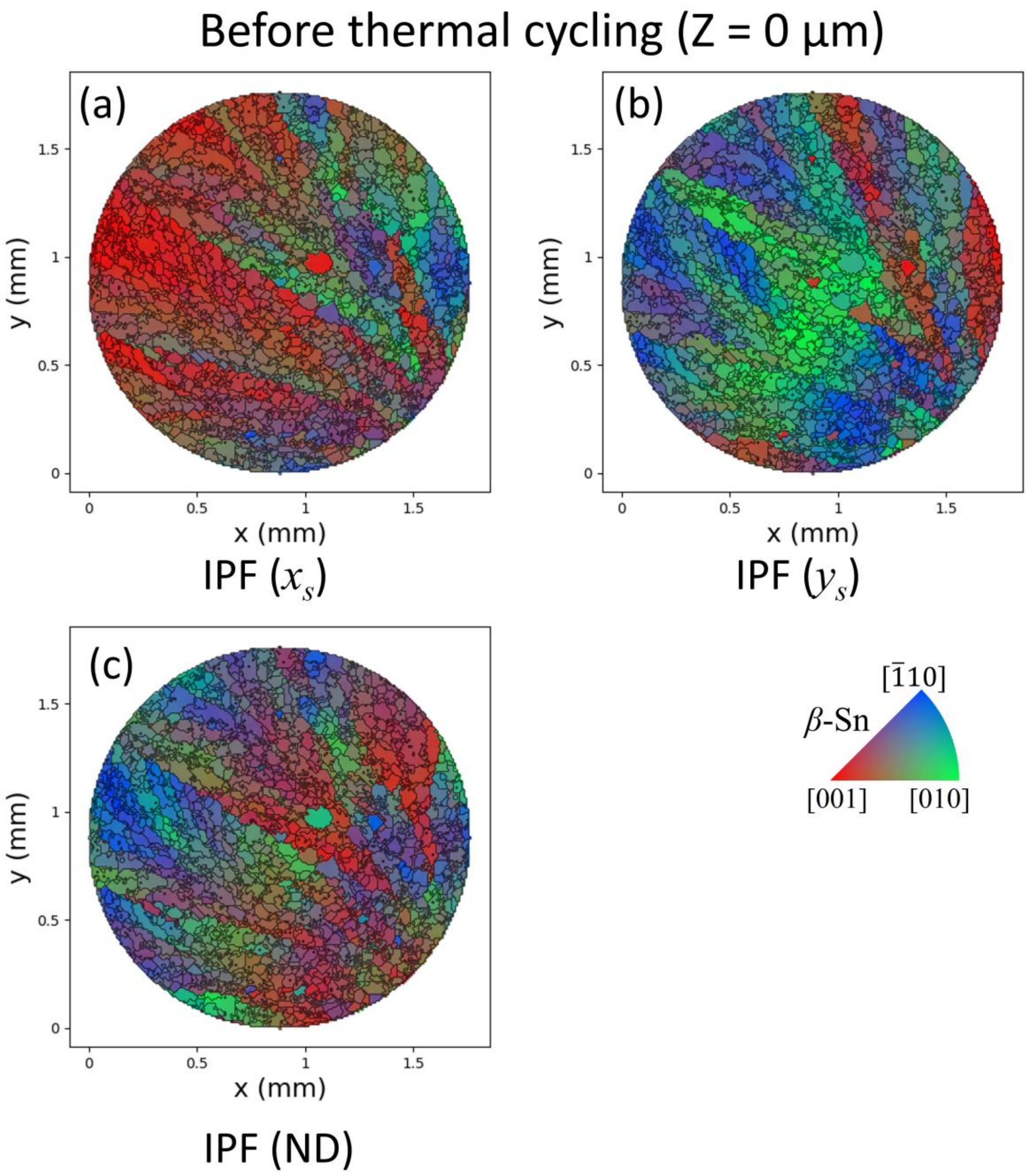

Fig. S9. IPF maps of the first specimen at z = 0μm before thermal cycling tests. (a-c) IPF (x_s), IPF (y_s), and IPF (ND) maps are illustrated with the grain boundaries of the 1° misorientation threshold.

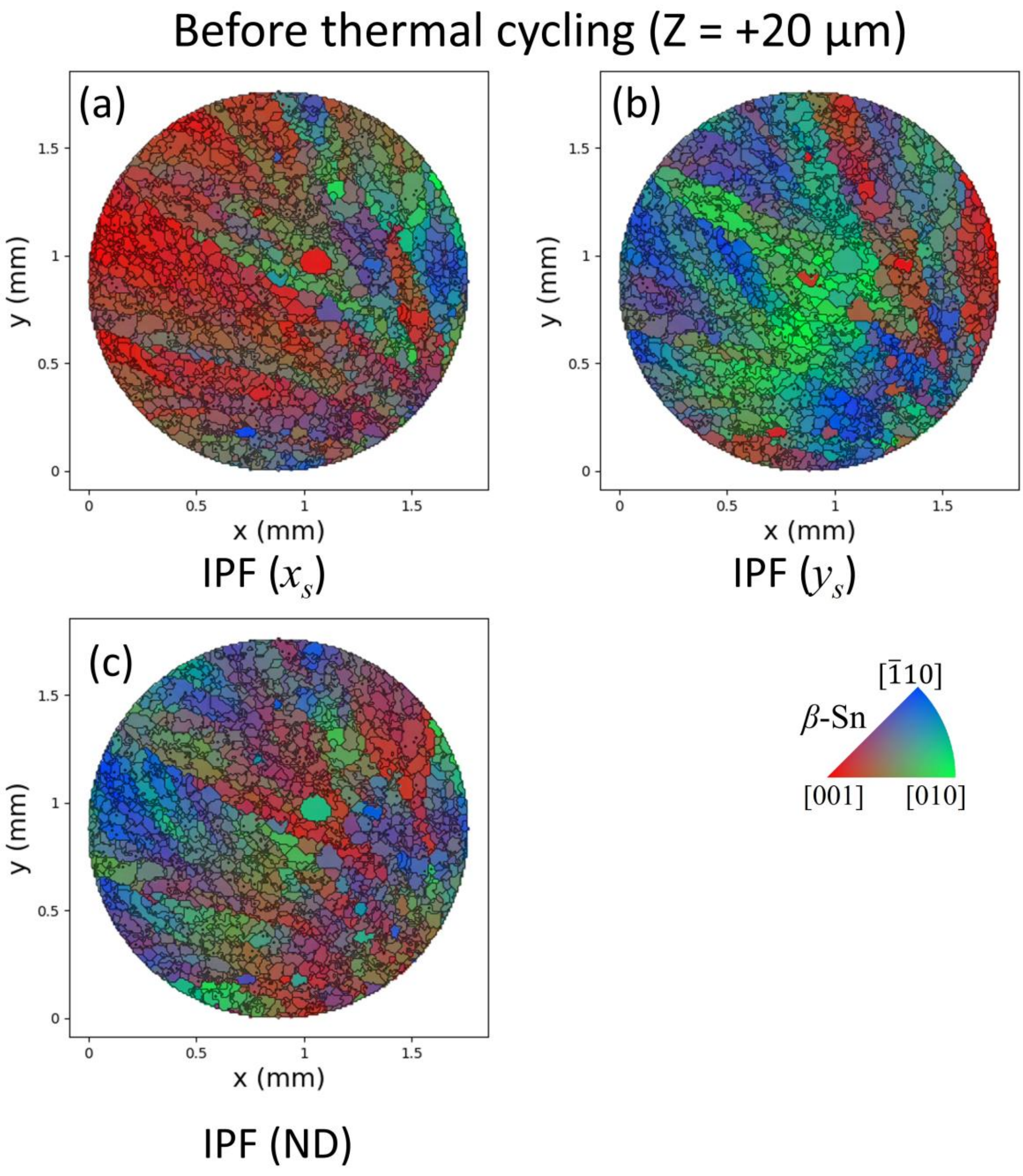

Fig. S10. IPF maps of the first specimen at z = +20μm before thermal cycling tests. (a-c) IPF (x_s), IPF (y_s), and IPF (ND) maps are illustrated with the grain boundaries of the 1° misorientation threshold.

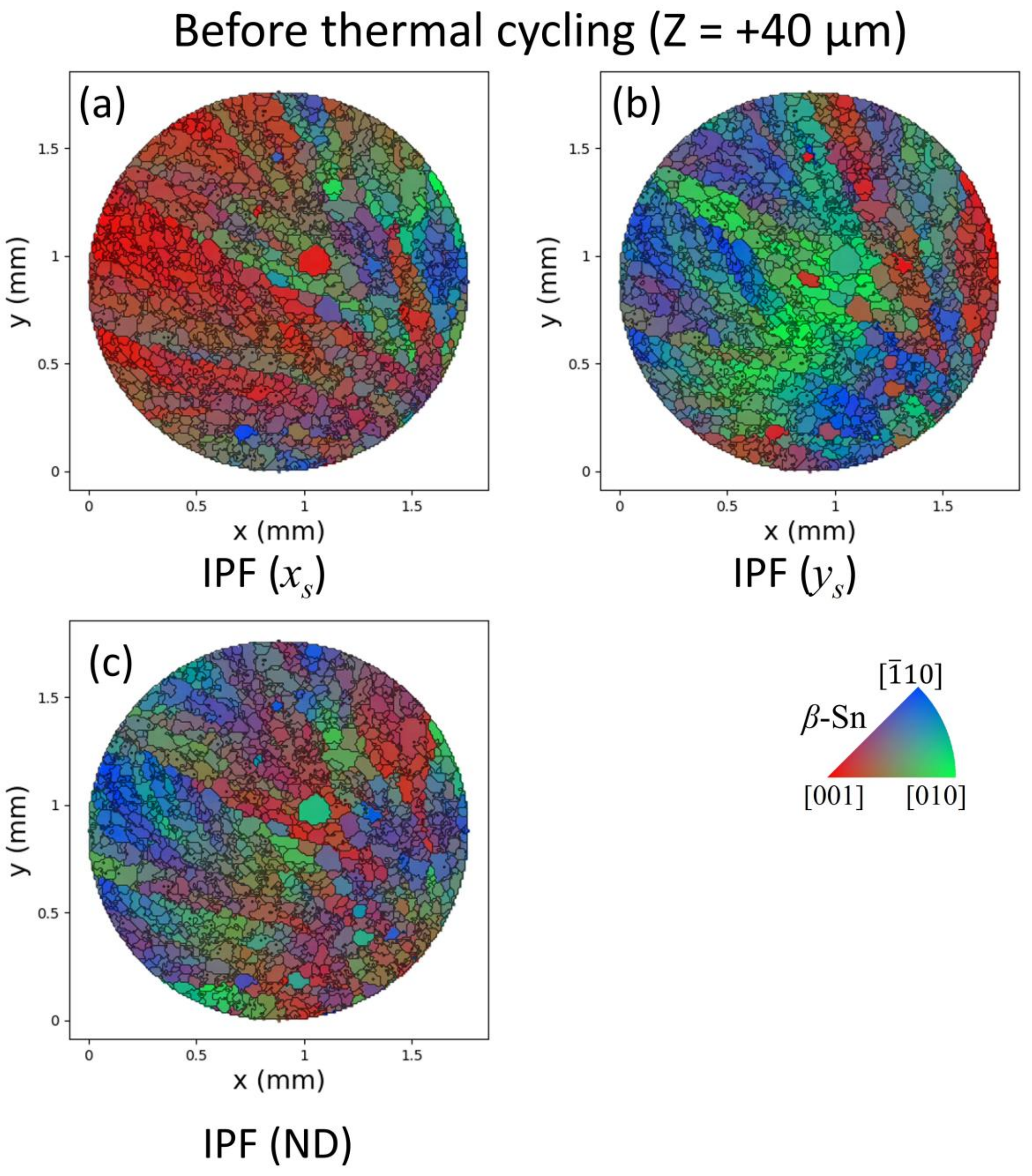

Fig. S11. IPF maps of the first specimen at z = +40μm before thermal cycling tests. (a-c) IPF (x_s), IPF (y_s), and IPF (ND) maps are illustrated with the grain boundaries of the 1° misorientation threshold.

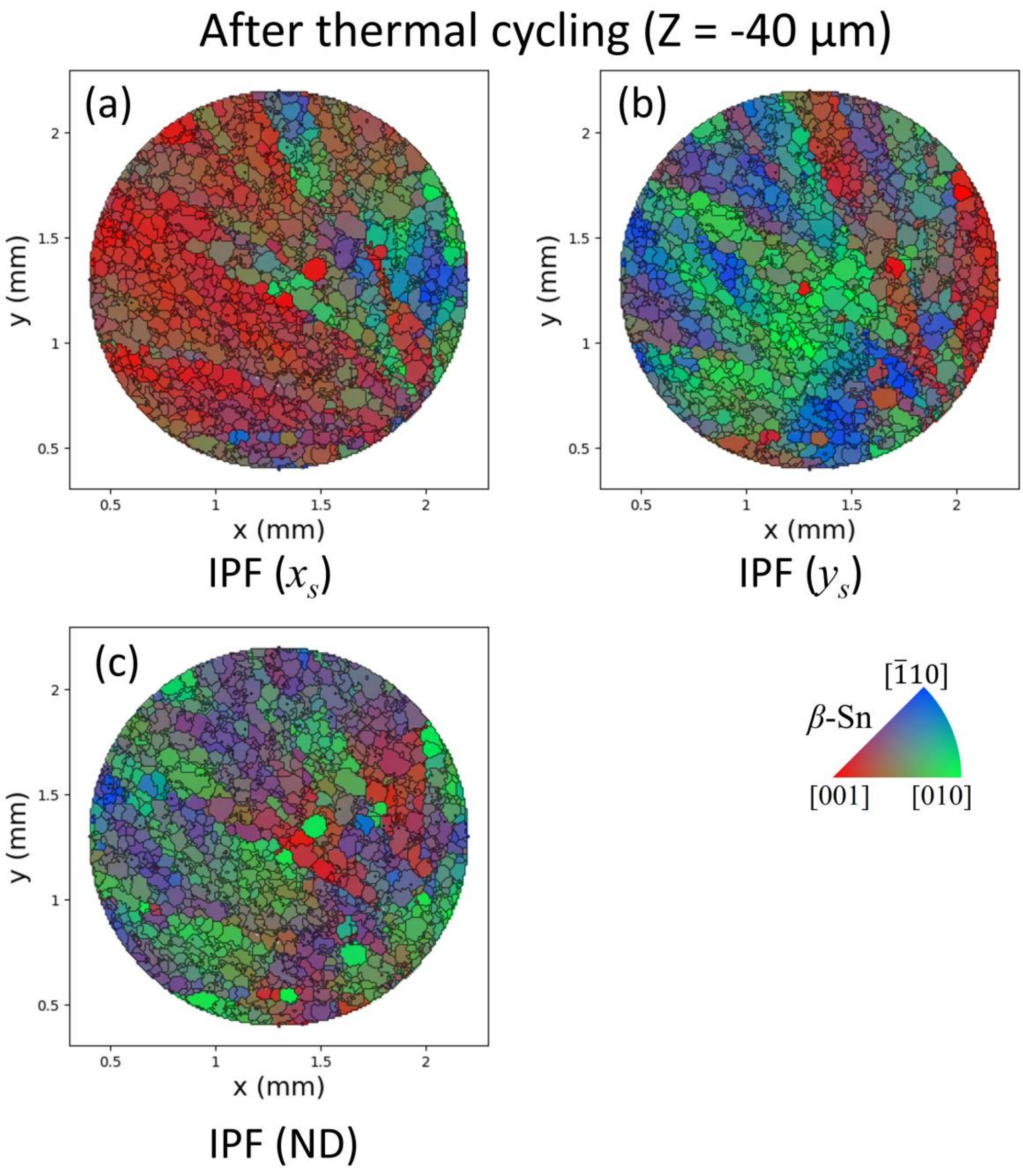

Fig. S12. IPF maps of the first specimen at z = -40μm after thermal cycling tests. (a-c) IPF (x_s), IPF (y_s), and IPF (ND) maps are illustrated with the grain boundaries of the 1° misorientation threshold.

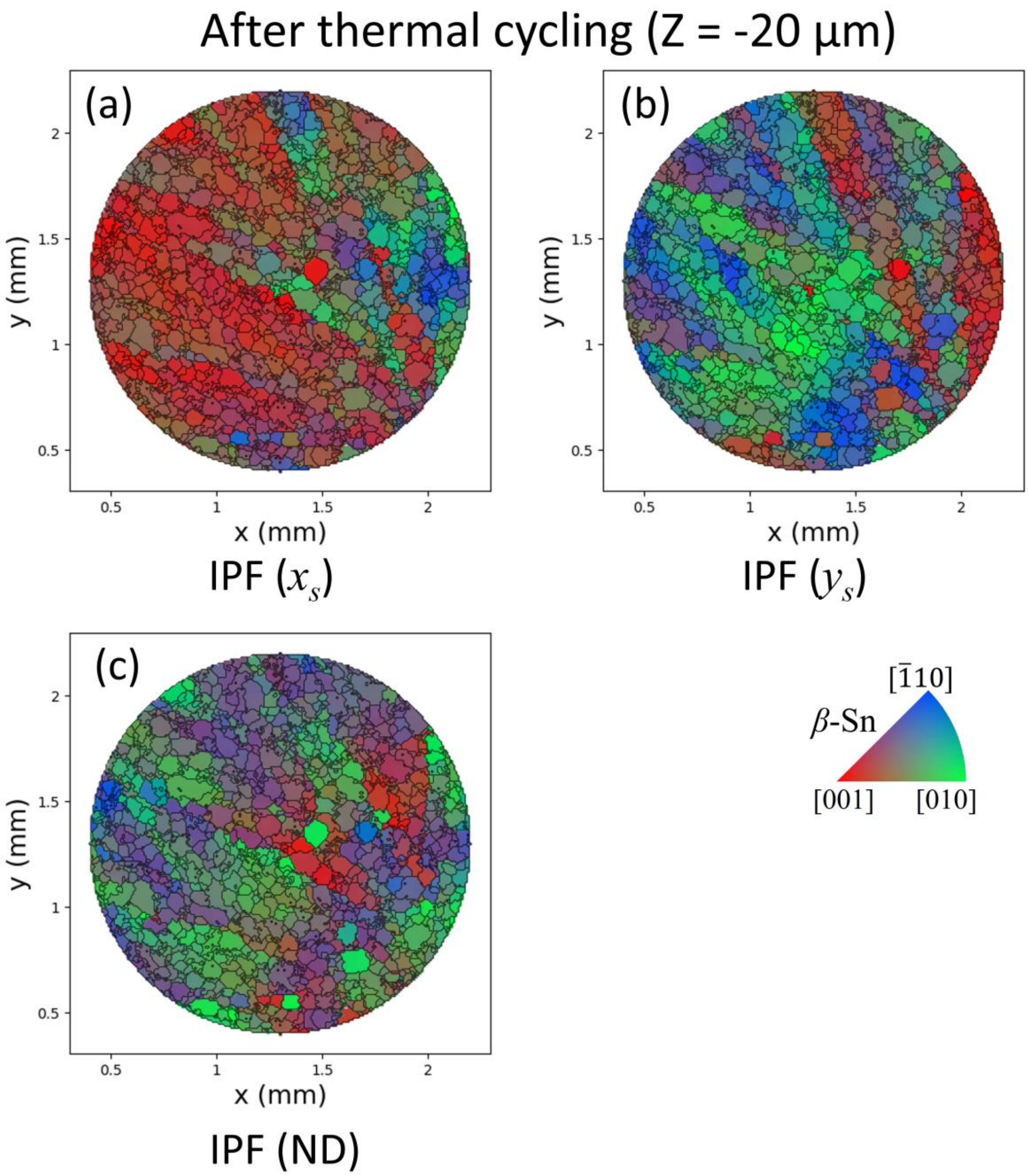

Fig. S13. IPF maps of the first specimen at z = -20μm after thermal cycling tests. (a-c) IPF (x_s), IPF (y_s), and IPF (ND) maps are illustrated with the grain boundaries of the 1° misorientation threshold.

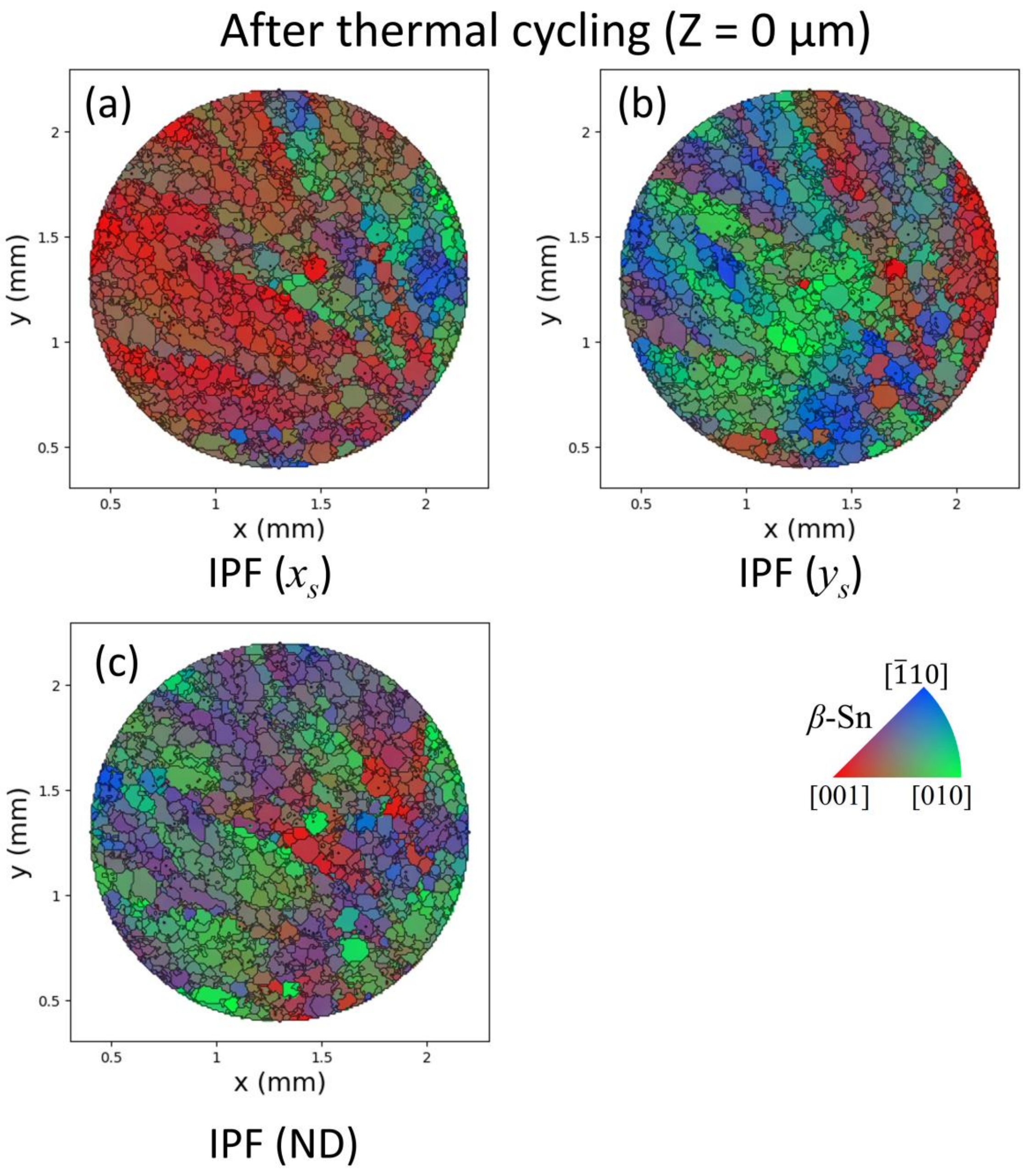

Fig. S14. IPF maps of the first specimen at z = 0µm after thermal cycling tests. (a-c) IPF (x_s), IPF (y_s), and IPF (ND) maps are illustrated with the grain boundaries of the 1° misorientation threshold.

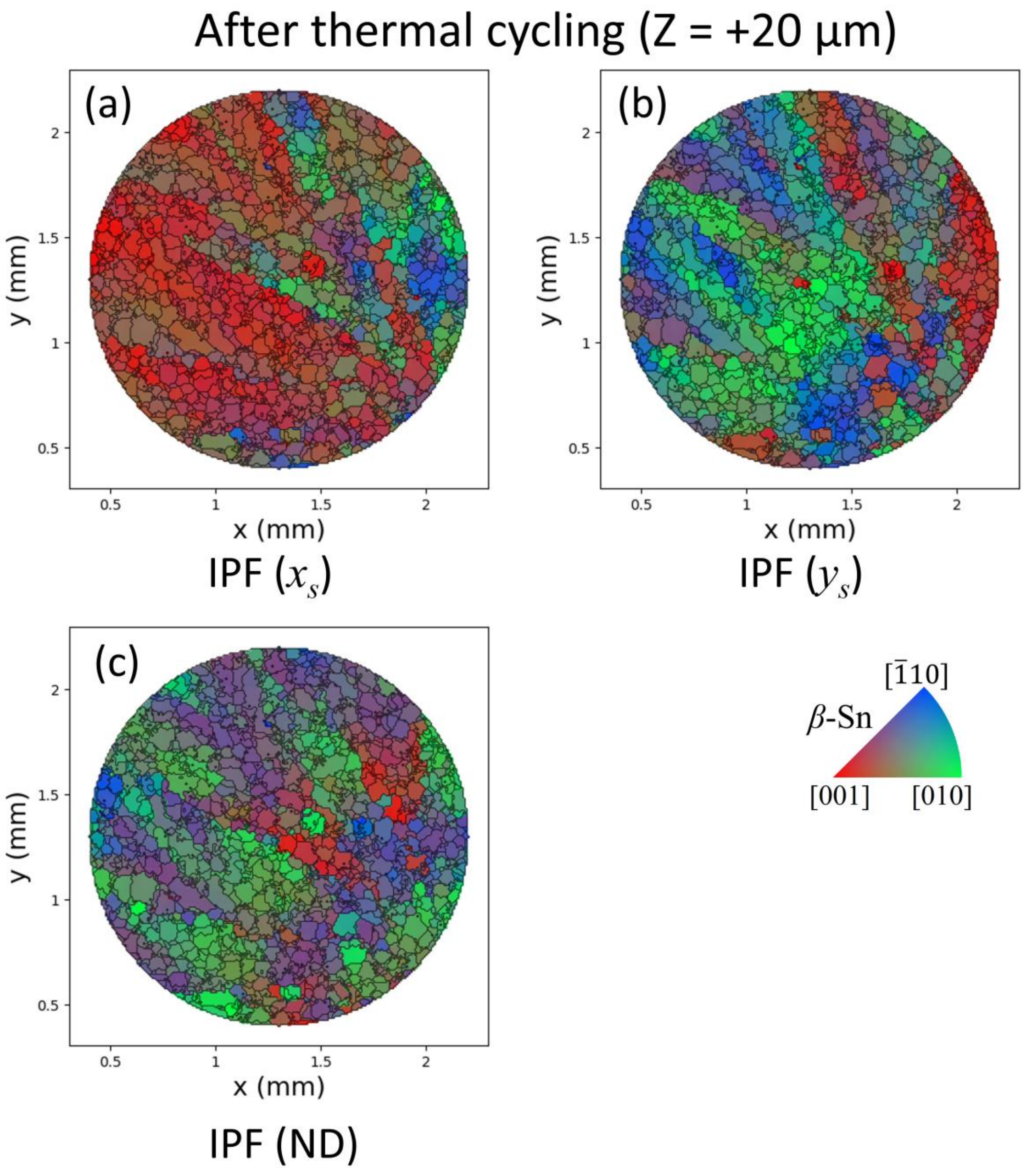

Fig. S15. IPF maps of the first specimen at z = +40µm after thermal cycling tests. (a-c) IPF (x_s), IPF (y_s), and IPF (ND) maps are illustrated with the grain boundaries of the 1° misorientation threshold.

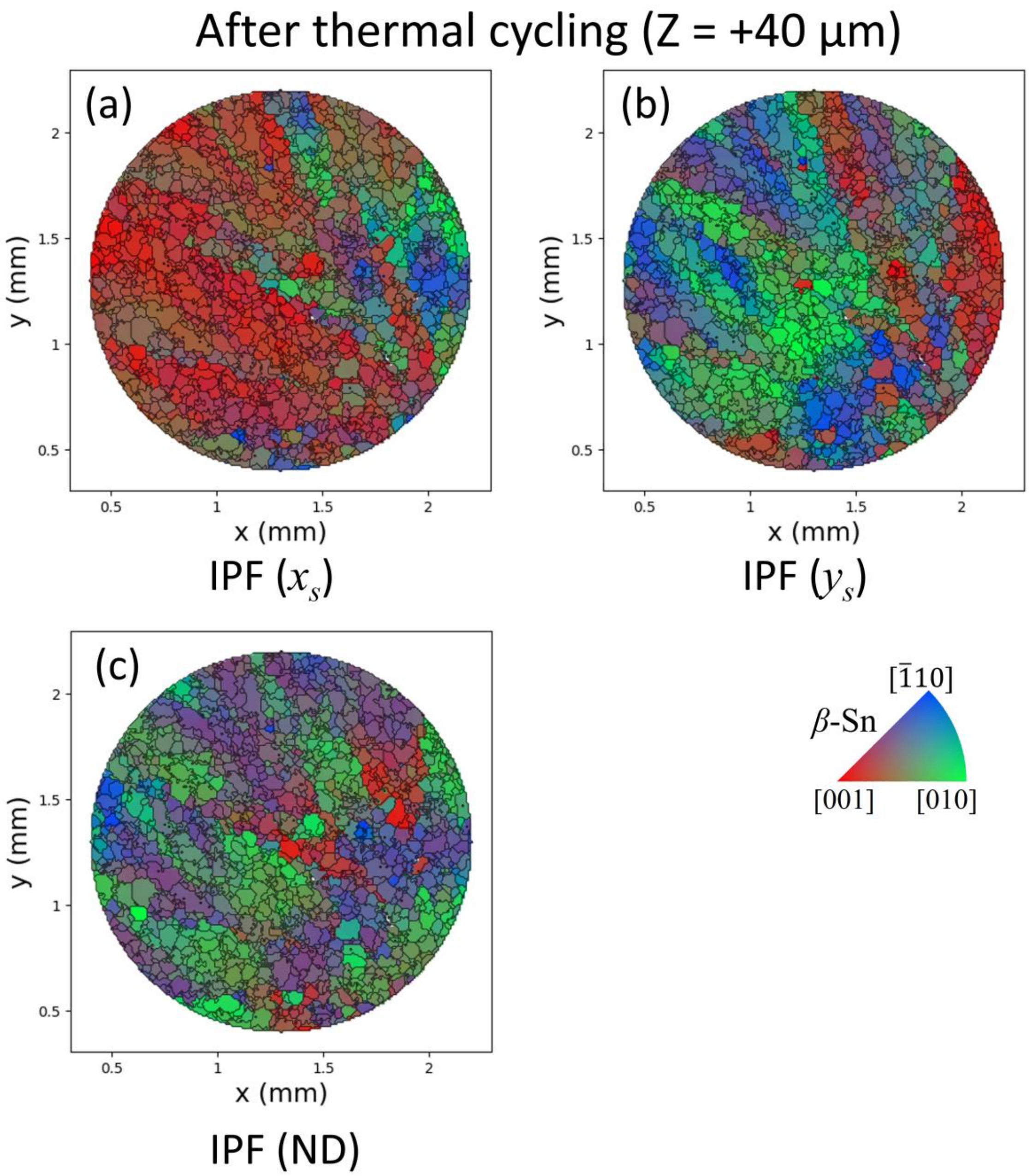

Fig. S16. IPF maps of the first specimen at z = +40μm after thermal cycling tests. (a-c) IPF (x_s), IPF (y_s), and IPF (ND) maps are illustrated with the grain boundaries of the 1° misorientation threshold.

Video. S1. 3D IPF (x_s, y_s, ND) maps of the first specimen before thermal cycling. The color in the video indicates the orientation in IPFs.

Video. S2. 3D IPF (x_s, y_s, ND) maps of the first specimen after thermal cycling. The color in the video indicates the orientation in IPFs.
